# Supplementary material for: Melt-quenched glass formation of a family of metal-carboxylate frameworks
Source: Nat Commun. 2024 Mar 6;15:2040. doi: 10.1038/s41467-024-46311-x (PMC10917788; doi:10.1038/s41467-024-46311-x)
Supplement: Supplementary file 1 — Supplementary Information [file 41467_2024_46311_MOESM1_ESM.pdf]

# Supplementary Information

## Melt-Quenched Glass Formation of a Family of Metal-Carboxylate Frameworks

Wen-Long Xue[1,6+], Guo-Qiang Li[1+], Hui Chen[1,7+], Yu-Chen Han[1], Li Feng[1], Lu Wang[1], Xiao-Ling Gu[1], Si-Yuan Hu[1], Yu-Heng Deng[1], Lei Tan[8], Martin T. Dove[9], Wei Li[3]*, Jiang-Wei Zhang[4]*, Hong-Liang Dong[5], Zhi-Qiang Chen[5], Wei-Hua Deng[2], Gang Xu[2]*, Guo Wang[1], Chong-Qing Wan[1,2,10]*

[1] Beijing Key Laboratory for Optical Materials and Photonic Devices, Department of Chemistry, Capital Normal University, Beijing 100048, China

E-mail: wancq@cnu.edu.cn

[2] State Key Laboratory of Structural Chemistry, Fujian Institute of Research on the Structure of Matter, Chinese Academy of Sciences, Fuzhou, Fujian 350002, China

E-mail: gxu@fjirsm.ac.cn

[3] School of Materials Science and Engineering & Tianjin Key Laboratory of Metal and Molecule-Based Material Chemistry, Nankai University, Tianjin 300350, China

E-mail: wl276@nankai.edu.cn

[4] College of Chemistry and Chemical Engineering, Inner Mongolia University, Hohhot 010021, China

E-mail: jwz@imu.edu.cn

[5] Center for High Pressure Science and Technology Advanced Research, Pudong, Shanghai 201203, China

[6] Anorganische Chemie, Fakultät für Chemie & Chemische Biologie, Technische Universität Dortmund, Otto-Hahn Straße 6, Dortmund 44227, Germany

[7] School of Chemistry and Chemical Engineering, Xi'an University of Architecture and Technology, Xi'an 710055, China

[8] School of Sciences, Wuhan University of Technology, Wuhan, Hubei 430070, China

[9] College of Computer Science, Sichuan University, Chengdu, Sichuan 610065, China

[10] Key Laboratory of Bioorganic Phosphorus Chemistry & Chemical Biology (Ministry of Education), Department of Chemistry, Tsinghua University, Beijing 100084, China

[+]These authors contributed equally to this work.

# Table of Contents

Supplementary Section 1. Characterization related to UiO-67, ZW-UiO-67 and their derivatives...2

Supplementary Section 2. Characterization related to ZW-DUT-5 and its derivatives. ..................26

Supplementary Section 3. Characterization related to ZW-UiO-68 and its derivatives. ..................27

Supplementary Section 4. Supplementary Tables. ..................31

Supplementary References..................35

## Supplementary Section 1. Characterization related to UiO-67, ZW-UiO-67 and their derivatives.

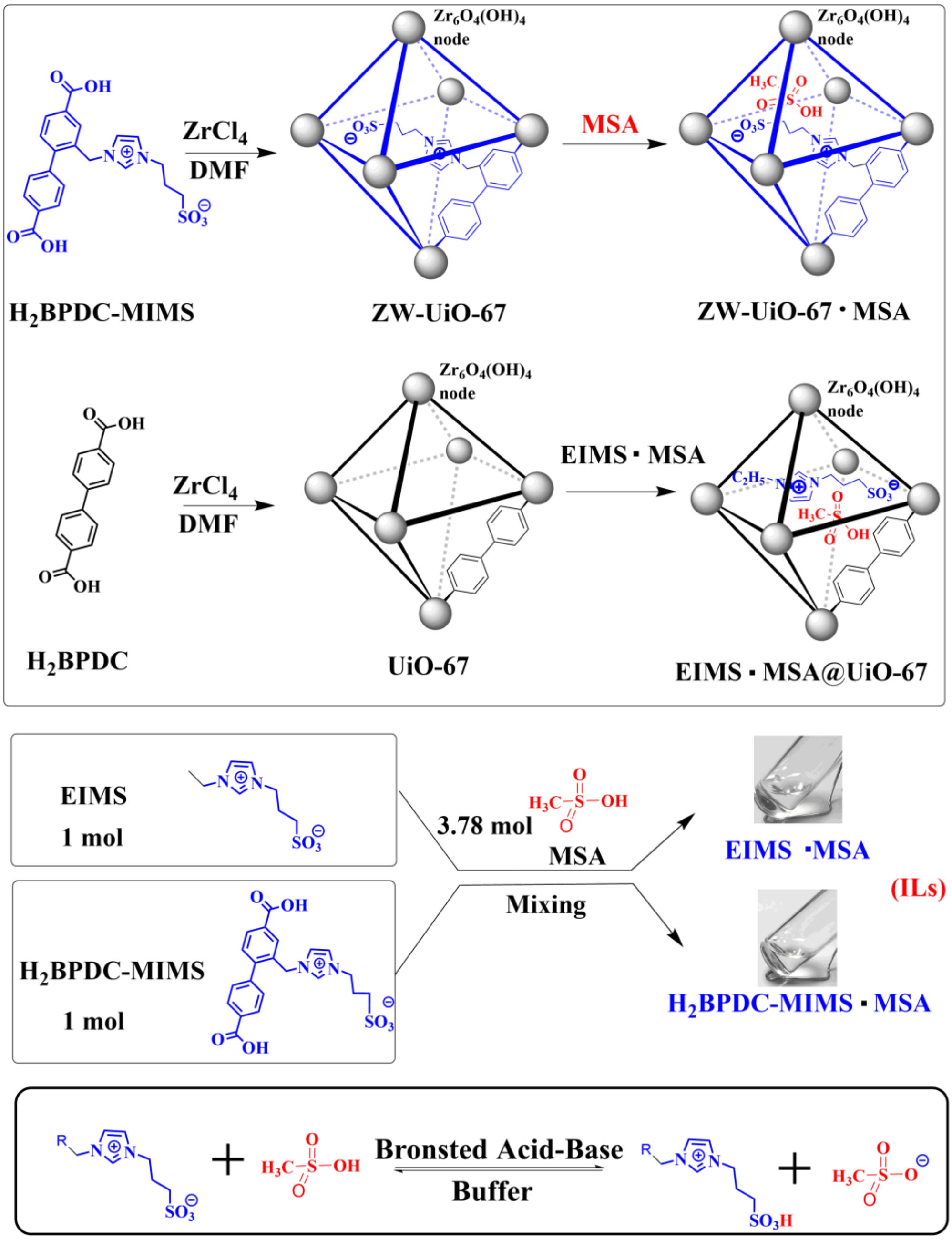


**Supplementary Fig. 1 | Representative synthetic illustration of ZW-UiO-67·MSA, counterpart EIMS·MSA@UiO-67 and acid-zwitterion ionic liquids (ILs)**. **ZW-UiO-67·0.5MSA**, other **ZW-UiO-67·HA** and ILs were obtained via the same procedure as that illustrated. The bottom presents the acid-zwitterion buffer with fast $H^+$ exchange within a Brønsted acidic ionic liquid[1]. The molar ratio of zwitterion EIMS (1-ethylimidazolium-3-propanesulfonate) to MSA (methylsulfonic acid) equals to 3.78:1, being same as that ratio of BPDC-MIMS:MSA within **ZW-UiO-67·MSA** and that **$H_2$BPDC-MIMS:MSA** in bulk ionic liquid **$H_2$BPDC-MIMS·MSA**. More information of HA (MSA, TFSA, TFA, ESA) or ILs are detailed and listed in Supplementary Table 2.

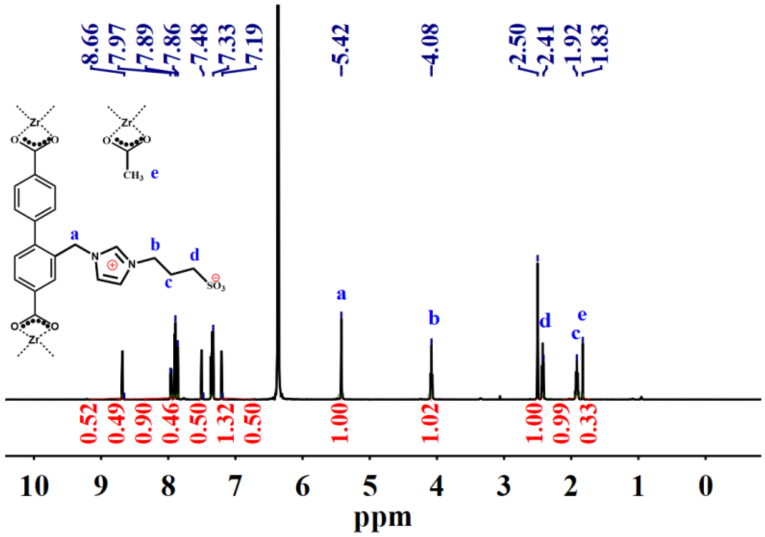


**Supplementary Fig. 2 |** $^{1}$H NMR of ZW-UiO-67 digested in deuterium chloride (6.33 ppm) and DMSO-d6 (2.50 ppm).

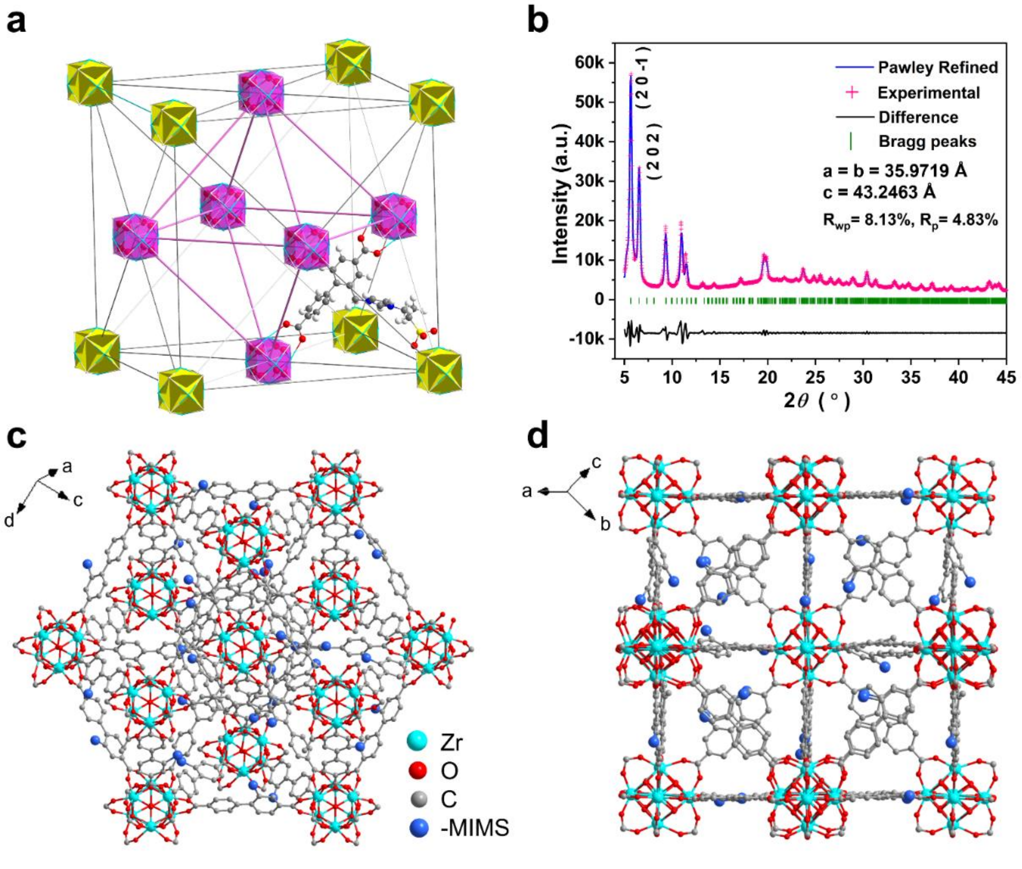


**Supplementary Fig. 3 | Modeled crystal structure of ZW-UiO-67. a**, Modeled structure of **ZW-UiO-67** in *R*3 space group, with link BPDC-MIMS rationalized as different color lines, with purple and gold polyhedron representing cluster $Zr_6O_6(OH)_6$ for clarity. **ZW-UiO-67** is isostructural MOF of UiO-67 (Supplementary Fig. 1), featuring tetrahedrons ($\varphi \approx 12$ Å) and octahedrons ($\varphi \approx 16$ Å) that share the triangular windows with 8 Å across[2]. **b**, PXRD pattern comparison of experiment (pink) and Pawley refined results (blue) in good agreement. **c**, (20-1), and **d**, (202) plane structure in **ZW-UiO-67** with the zwitterionic MIMS groups presented as blue balls for clarity.

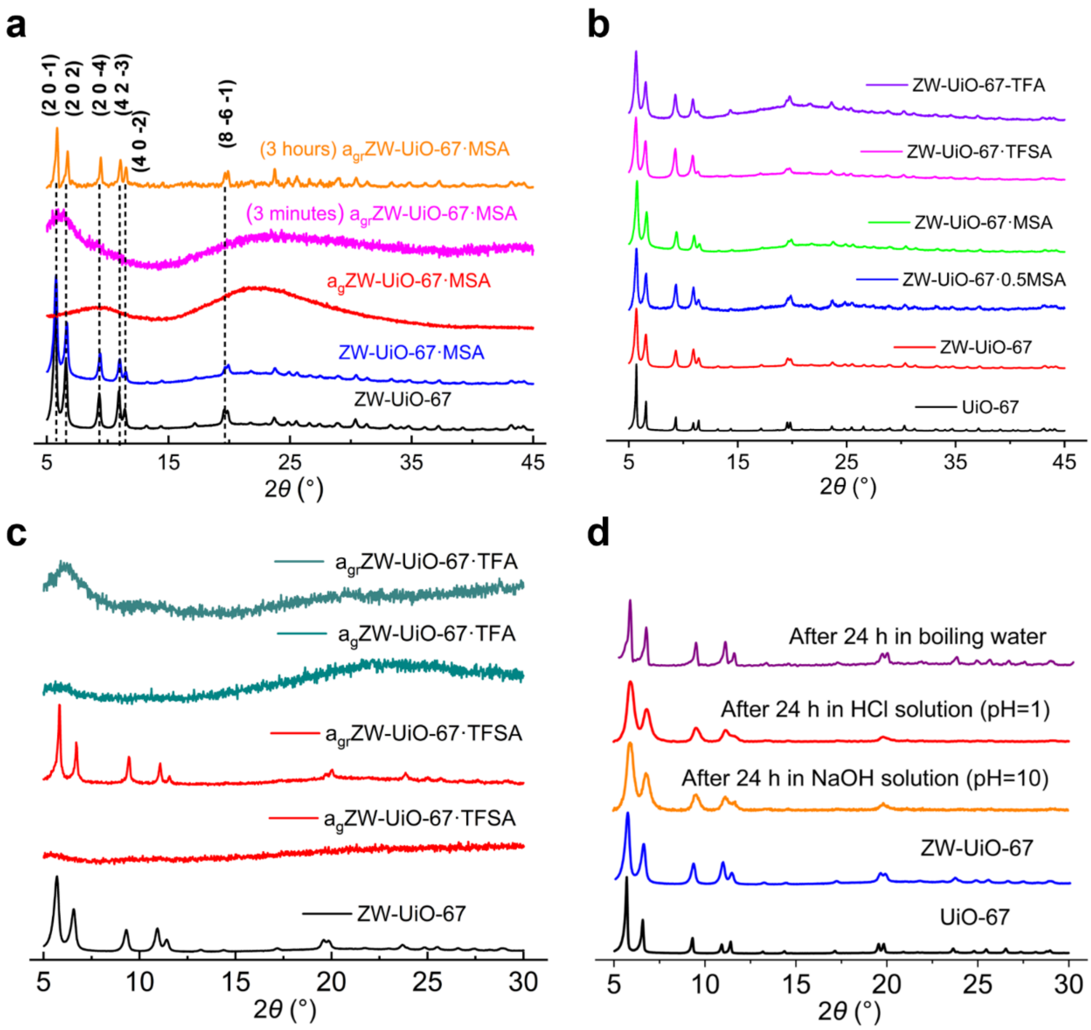


**Supplementary Fig. 4 | PXRD patterns of ZW-UiO-67 and derivatives. a**, Patterns of **ZW-UiO-67**, **ZW-UiO-67·MSA**, **a$_g$ZW-UiO-67·MSA**, and **a$_{gr}$ZW-UiO-67·MSA** produced via 3 minutes soaking **a$_g$ZW-UiO-67·MSA** in methanol at room temperature and 3 hours refluxing in comparison. **b**, Compared patterns of crystalline UiO-67, **ZW-UiO-67**, **ZW-UiO-67·HA** and **ZW-UiO-67·0.5MSA** (HA = MSA, TFSA, TFA). **c**, Patterns of **a$_g$ZW-UiO-67·HA** and **a$_{gr}$ZW-UiO-67·HA** in comparison (HA = TFSA, TFA). Diffraction vanished for all **a$_g$ZW-UiO-67·HA**, while peaks at low 2$\theta$ degree such as that for (20-1) and (202) recovered in **a$_{gr}$ZW-UiO-67·TFSA** but broadened in **a$_{gr}$ZW-UiO-67·TFA** upon solvent stimulation. **d**, Remained PXRD patterns of **ZW-UiO-67** at different conditions. Broadened and weakened peaks as compared to that of pristine **ZW-UiO-67** may be due to their crystal morphology changed.

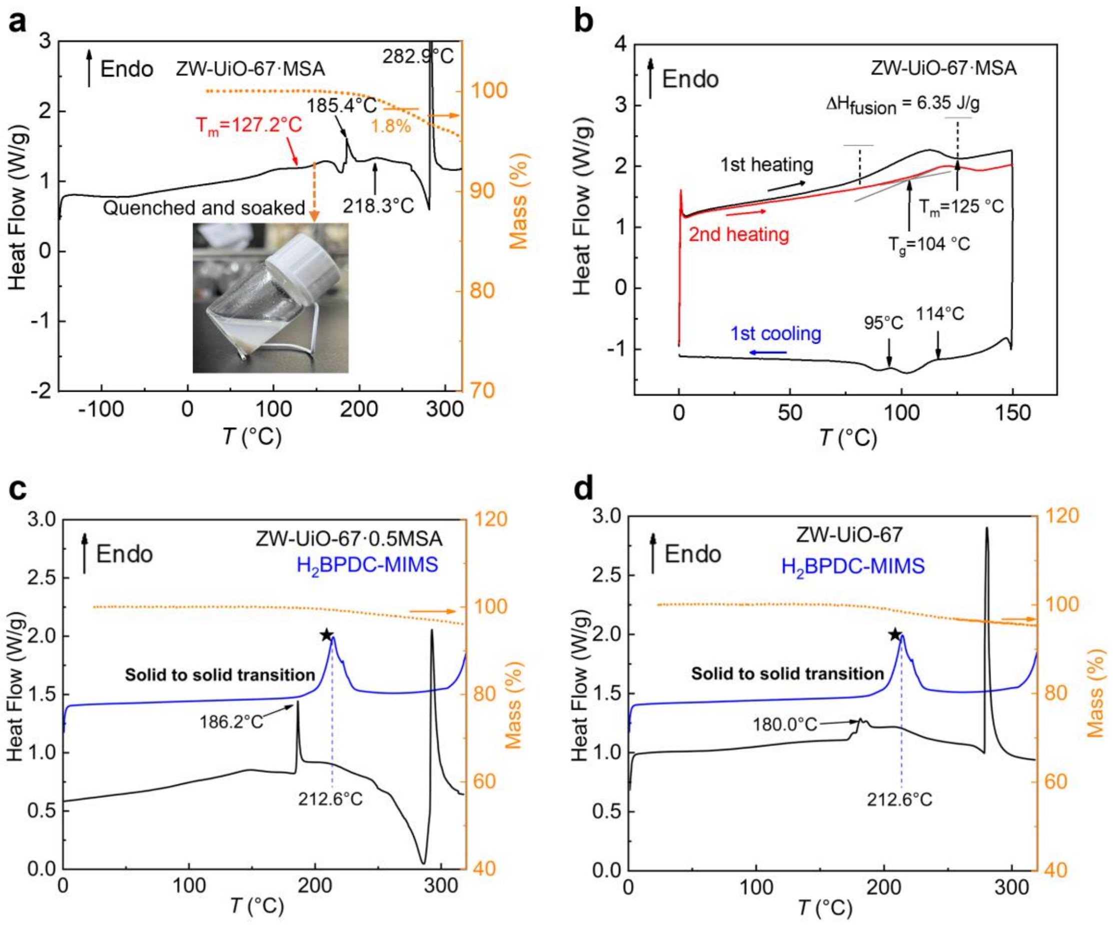


**Supplementary Fig. 5 | DSC curves of the linker $H_2$BPDC-MIMS and ZW-UiO-67 with and without incorporation of MSA. a**, Up-scan curve of **ZW-UiO-67·MSA** from –150 to 320 °C, with thermogravimetric analysis (TGA) trace shown as yellow dashed line, being consistent with Fig.1a. Inset photography is the soaking of melt-quenched solids (from 130 °C) in methanol for 3 minutes at RT, obtaining precipitates with weaken Bragg scattering as shown in Supplementary Fig. 4a. **b**, Special cyclic DSC curve of **ZW-UiO-67·MSA** from 0 °C to 150 °C shown with $T_m$, $T_g$ phase transition temperatures and fusion enthalpy. **c-d**, Compared curves of **ZW-UiO-67·0.5MSA** and **ZW-UiO-67** (0 ~ 320 °C) (up-scan) along with TGA traces of yellow dashed lines (25 ~ 800 °C). Plot of ligand $H_2$BPDC-MIMS (blue line) is presented as a reference, with asterisk inferring the phase transition and the dashed-lines guiding that similar event occurred for **ZW-UiO-67·0.5MSA** and **ZW-UiO-67** at this temperature. See also the detailed discussion and analysis of DSC in Method section.

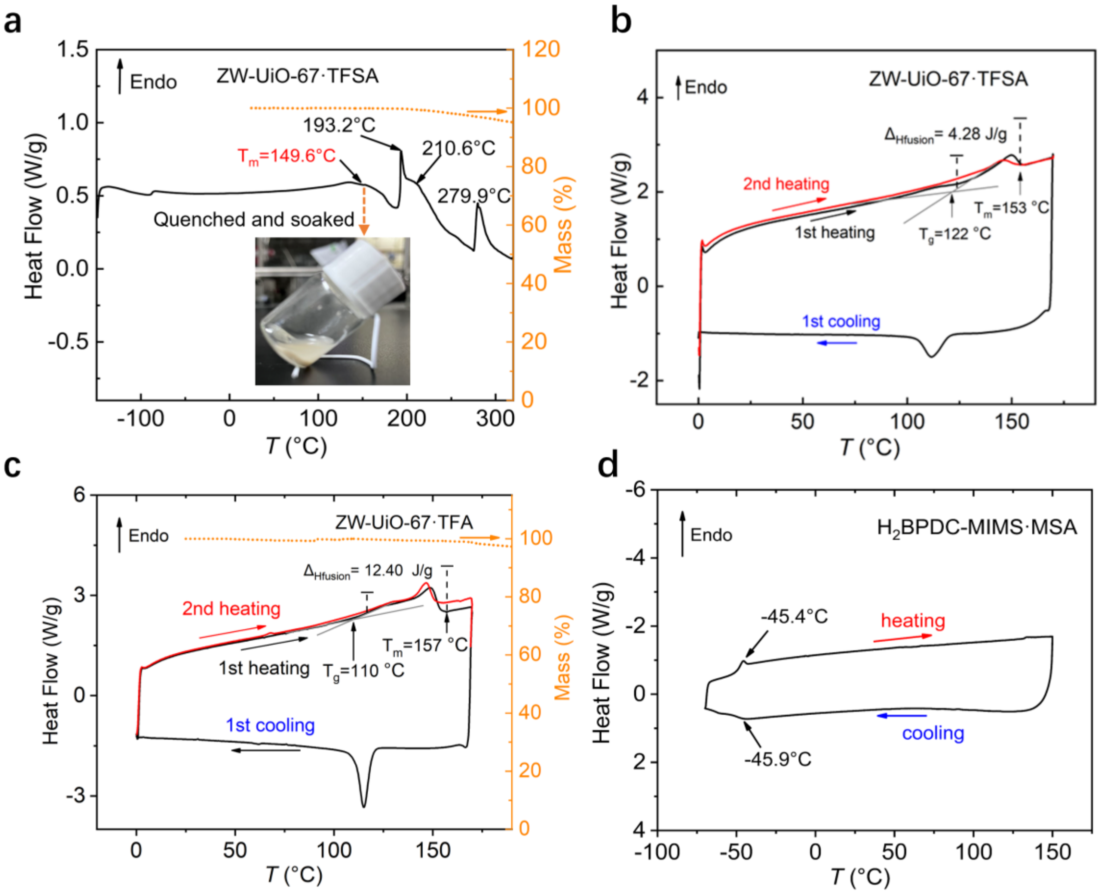


**Supplementary Fig. 6 | DSC curves of ZW-UiO-67·TFSA, ZW-UiO-67·TFA and ionic liquid $H_2$BPDC-MIMS·MSA. a**, Curve of **ZW-UiO-67·TFSA** within -150~320 °C range (up scan), with the TGA plot of yellow dashed line. Inset photography shows the soaking of melt-quenched solids (from 150 °C) in methanol for 3 min, resulting the precipitates of **$a_{gr}$ZW-UiO-67·TFSA**. **b**, Cyclic DSC of **ZW-UiO-67·TFSA** within 0~170 °C, with melting temperature ($T_m$), glass transition temperature ($T_g$) and fusion enthalpy indicated. **c**, Cyclic curve of **ZW-UiO-67·TFA** (0~170 °C), with $T_m$, $T_g$ temperature and fusion enthalpy indicated. TGA trace is shown as yellow dashed line. **d**, Heat-cool DSC curve of ionic liquid **$H_2$BPDC-MIMS·MSA** in comparison, showing the class melting and crystallization events in subzero region. See also relevant and detailed discussion for the DSC in Method section and main text.

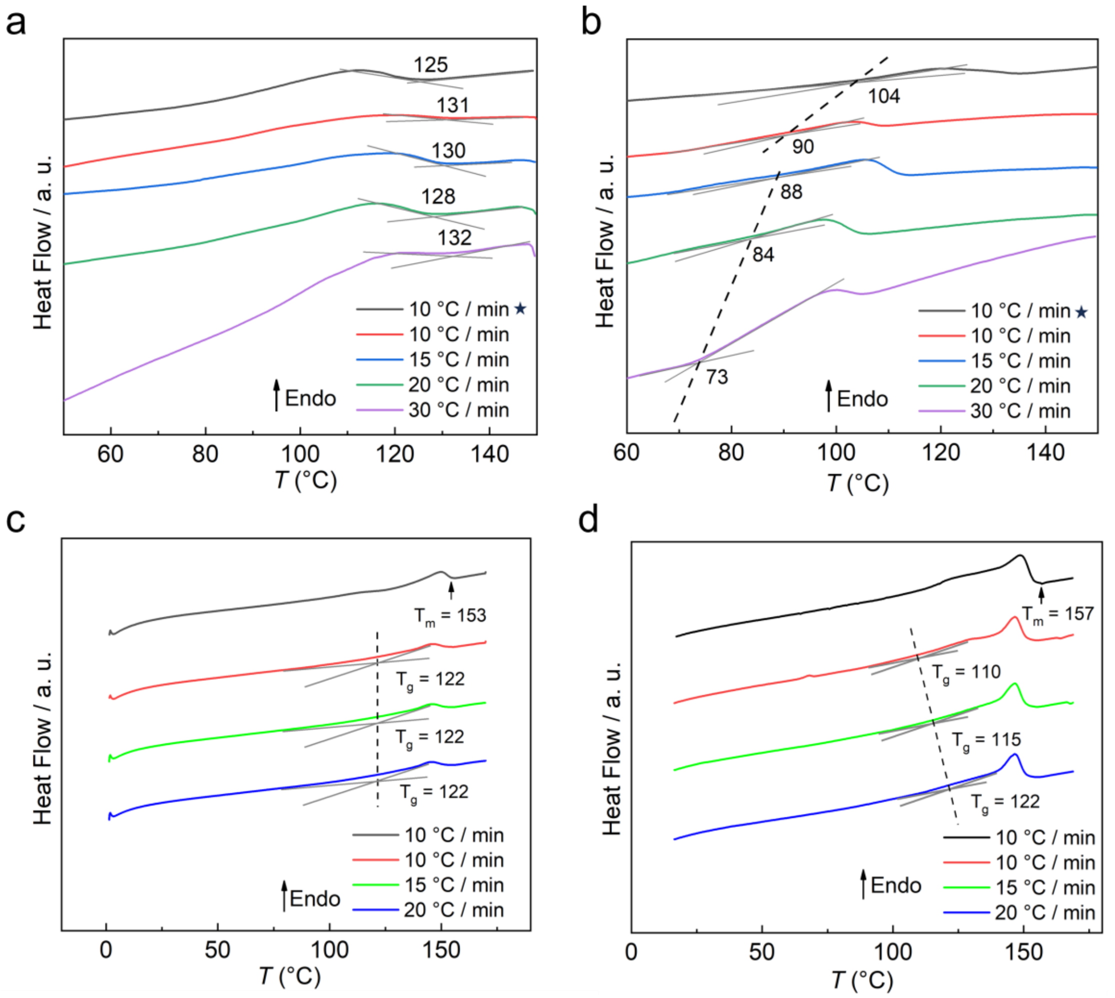


**Supplementary Fig. 7 | Different flowing gas and/or heating rate for DSC of ZW-UiO-67·MSA and ZW-UiO-67·TFSA**. **a**, Repeated DSC measurements by using different samples to show the effect of heating rate and different batch on the $T_m$, and **b**, the effect on $T_g$ of **ZW-UiO-67·MSA**. Asterisk infer the gas flowing rate of 80 mL/min that is different from those of all others (50 mL/min). **c**, Repeated cyclic DSC plot from 0 °C to 170 °C and the following down to 0 °C, and then up scan to 170 °C (black line) with heating rate of 10 °C/min for **ZW-UiO-67·TFSA**. The red and turquoise plots are the second and third cyclic data with different heating rates, while the blue line is the last up scan of the **a$_g$ZW-UiO-67·TFSA**. **d**, comparison of four up scan plots for **ZW-UiO-67·TFA** as that in panel **c**.

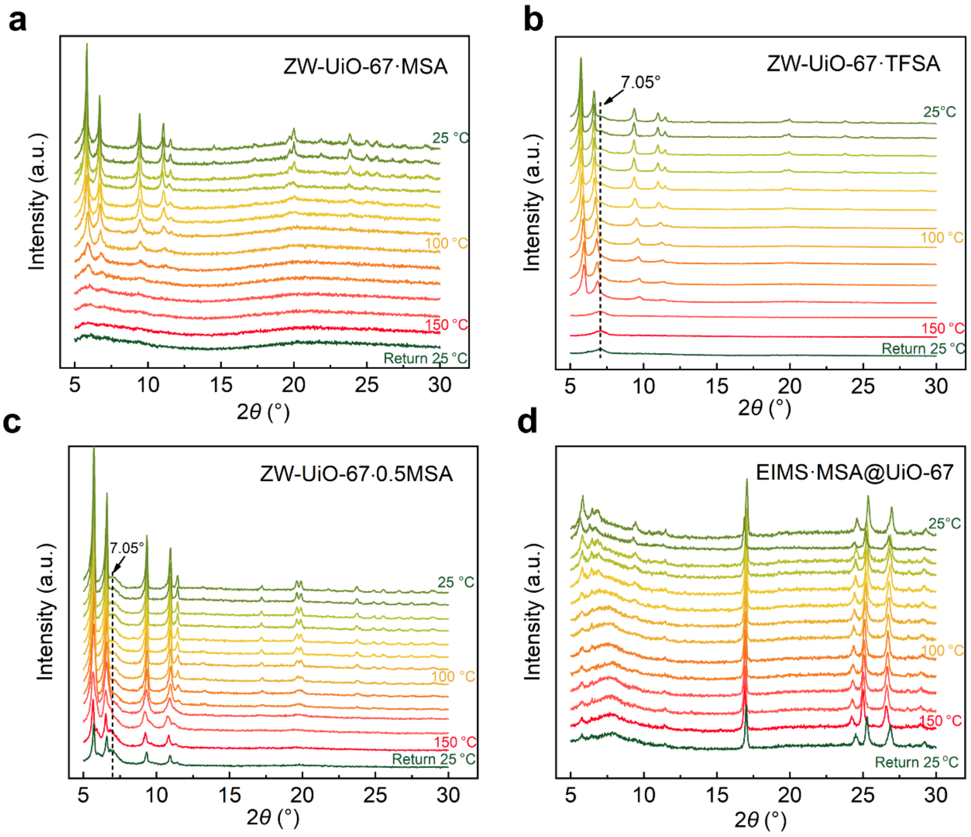


**Supplementary Fig. 8 | Varied-temperature PXRD patterns from 25 °C to 150 °C and then back to 25 °C. a**, **ZW-UiO-67·MSA. b**, **ZW-UiO-67·TFSA**. **c**, **ZW-UiO-67·0.5MSA. d**, **ILs@UiO-67**. The broad hump at $2\theta = 7.05°$ of **b-c**, which remains at 150 °C, is due to diffraction of Kapton from the instrument (Method) and not the samples. The weakening of the low-angle peaks in panel **d** may be due to the complete homogenization of ILs@UiO-67 resulted from high temperature heating.

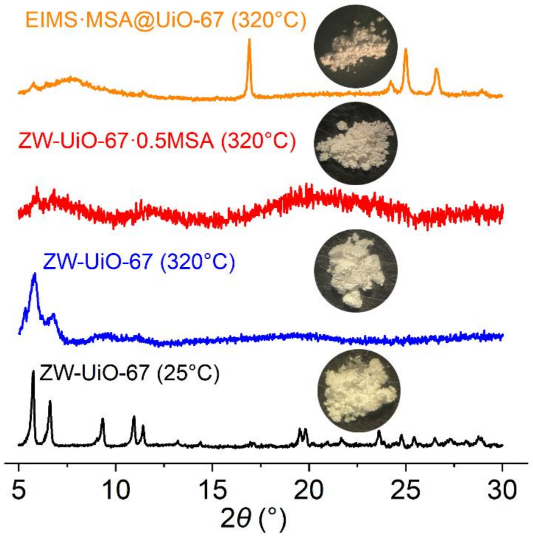


**Supplementary Fig. 9 | Compared PXRD patterns at 320 °C**. Patterns of **ZW-UiO-67**, **ZW-UiO-67·0.5MSA** and **EIMS·MSA@UiO-67** at 320 °C under $N_2$ atmosphere, and that of **ZW-UiO-67** at 25 °C as reference. Insets show the unchanged morphology of each sample.

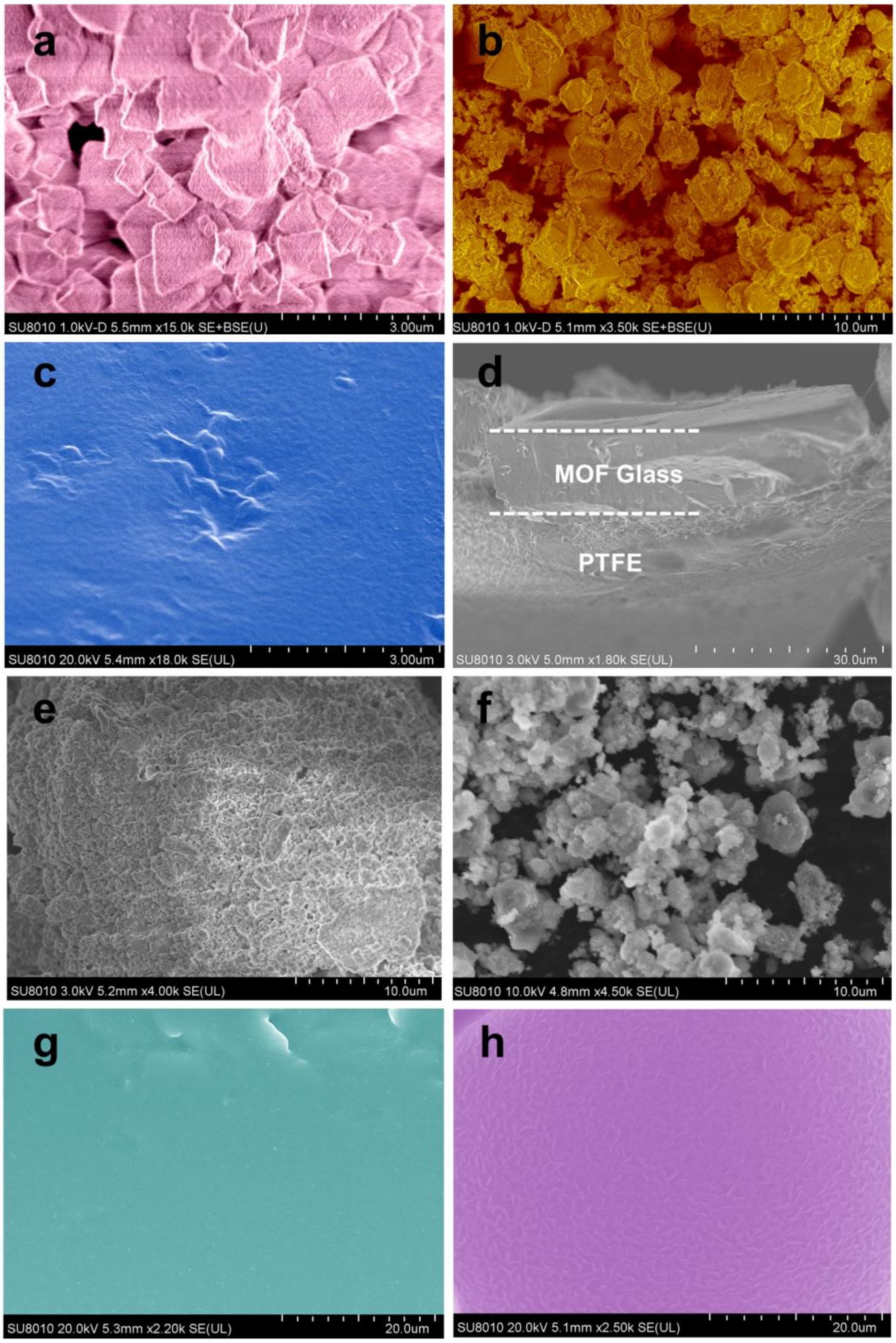


**Supplementary Fig. 10 | SEM images**. **a**, **ZW-UiO-67**. **b**, **ZW-UiO-67·MSA**. **c**, **$a_g$ZW-UiO-67·MSA**. **d**, Cross-sectional image of **$a_g$ZW-UiO-67·MSA**. PTFE polymer is used as support. **e**, **$a_{gr}$ZW-UiO-67·MSA** obtained via soaking in methanol with stirring at room temperature. **f**, **$a_{gr}$ZW-UiO-67·MSA** resulted from refluxing methanol. **g, $a_g$ZW-UiO-67·TFSA. h, $a_g$ZW-UiO-67·TFA**. The different false colors are shown for clarity.

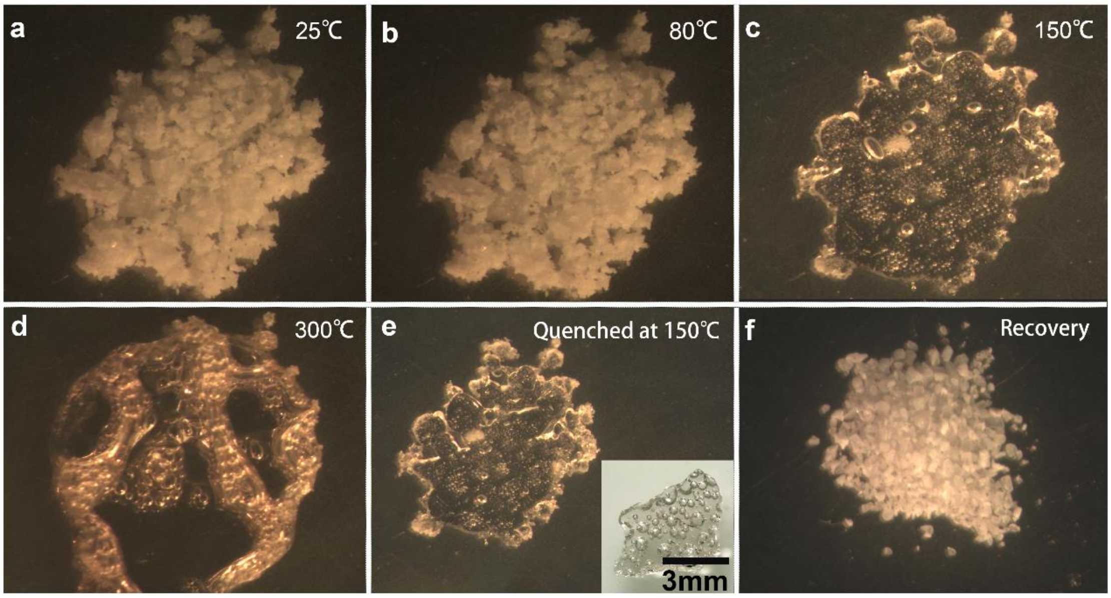


**Supplementary Fig. 11 | In-situ optical photography of ZW-UiO-67·MSA at different conditions**. **a-d**, Images of **ZW-UiO-67·MSA** at different temperatures. **e**, Image of glass **$a_g$ZW-UiO-67·MSA** (quenched from 150 °C) with inset of microscopic photography. **f**, Image of **$a_{gr}$ZW-UiO-67·MSA**. The bubbles in glass suggest a serious flow[3] as that shown in Video (SI).

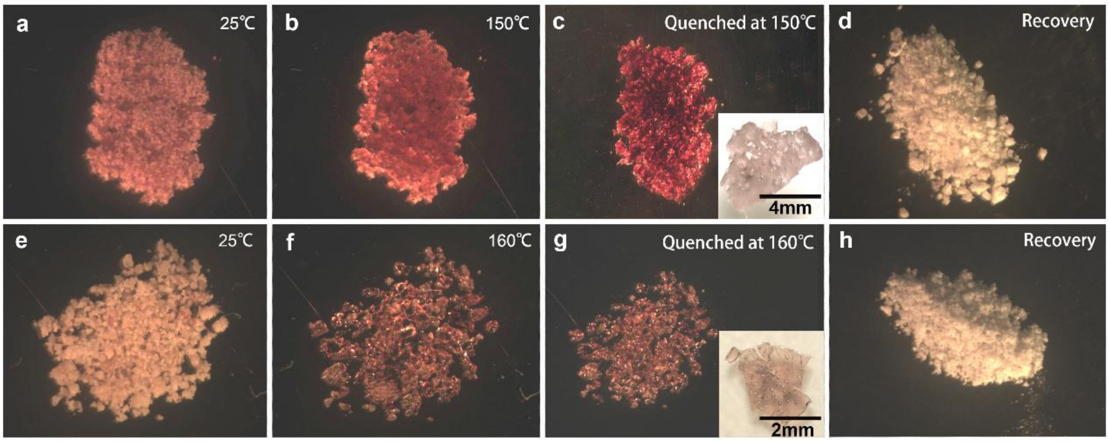


**Supplementary Fig. 12 | In-situ optical photographys of ZW-UiO-67·TFSA and ZW-UiO-67·TFA at different conditions**. **a-b**, Image of **ZW-UiO-67·TFSA** at 25 °C and 150 °C. **c**, Image of **$a_g$ZW-UiO-67·TFSA** (quenched from 150 °C) with inset of microscopic photography. **d**, Image of **$a_{gr}$ZW-UiO-67·TFSA**. **e-h**, Compared images for **ZW-UiO-67·TFA** at different conditions.

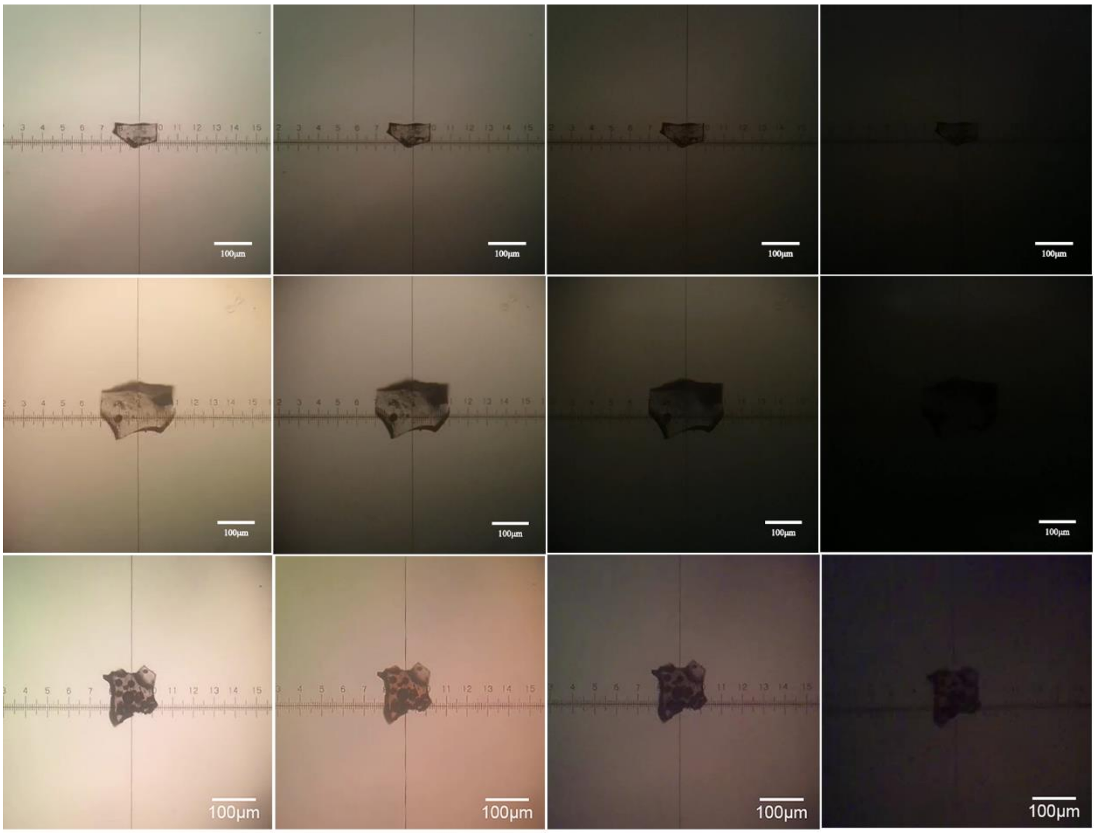


**Supplementary Fig. 13 | Polarized light microscopic images of $a_g$ZW-UiO-67·HA. Top**: Microscopy images of **$a_g$ZW-UiO-67·MSA** from parallel (left, light) to crossed polarized positions (right, dark). **Middle**: **$a_g$ZW-UiO-67·TFSA**. **Bottom**: **$a_g$ZW-UiO-67·TFA**.

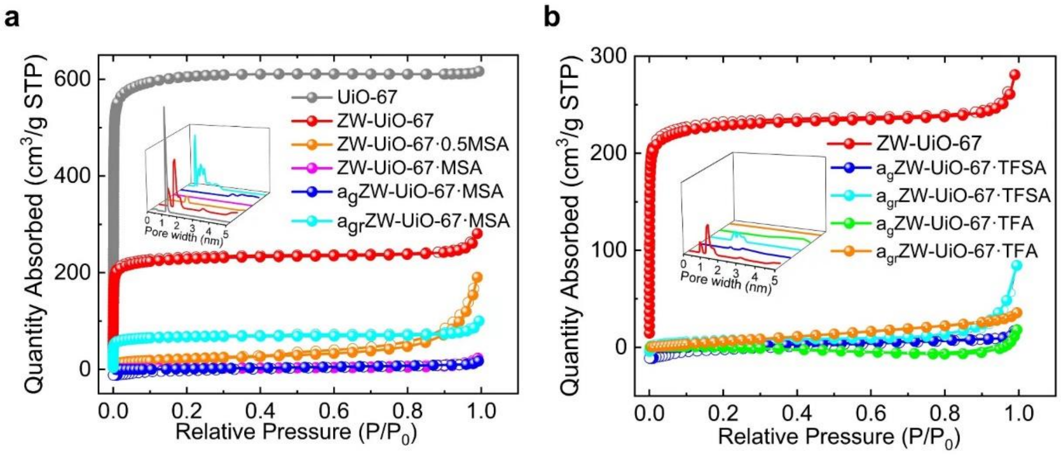


**Supplementary Fig. 14 | $N_2$ absorption-desorption isotherms and pore size distribution of parent UiO-67, ZW-UiO-67 and derivatives. a**, Isotherms at 77 K of UiO-67, **ZW-UiO-67**, **ZW-UiO-67·0.5MSA**, **ZW-UiO-67·MSA**, **$a_g$ZW-UiO-67·MSA** and **$a_{gr}$ZW-UiO-67·MSA** (product of reflux in methanol). **b**, these of **$a_g$ZW-UiO-67·TFSA/TFA** and **$a_{gr}$ZW-UiO-67·TFSA/TFA** (product after 3 minutes soaked in methanol). Pore distributions are shown as inset graphics.

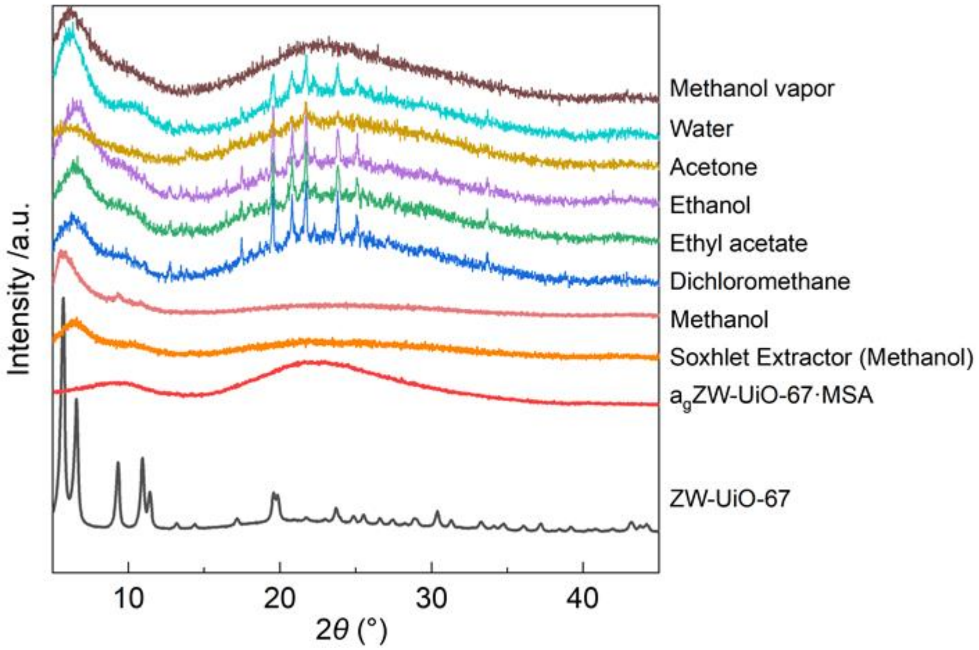


**Supplementary Fig. 15 | PXRD patterns of $a_{gr}$ZW-UiO-67·MSA after solvent stimulations in comparison.** Products were obtained via soaking **$a_g$ZW-UiO-67·MSA** in different solvents with stirring at room temperature for 20 minutes. Compared to the patterns of the sample soaked of 3 minutes in methanol (Supplementary Fig. 4), peaks at high $2\theta$ degree appear for those of 20 minutes in various solvents. Methanol vapor stimulation is done by exposing sample to vapor for 30 minutes. Soxhlet Extractor method with methanol as solvent is used to rule out the possibility of **$a_{gr}$ZW-UiO-67·MSA** from the gradually dissolving **$a_g$ZW-UiO-67·MSA** and recrystallization. Except for the effect of different solvents, the different crystalline phases of $a_{gr}$ZW-UiO-67-MISA can also be explained by the effect of different batches, including distinct composites obtained via the incipient wetness technique, uncertain portion departure of MSA and different conditions such as with or without stirring etc.

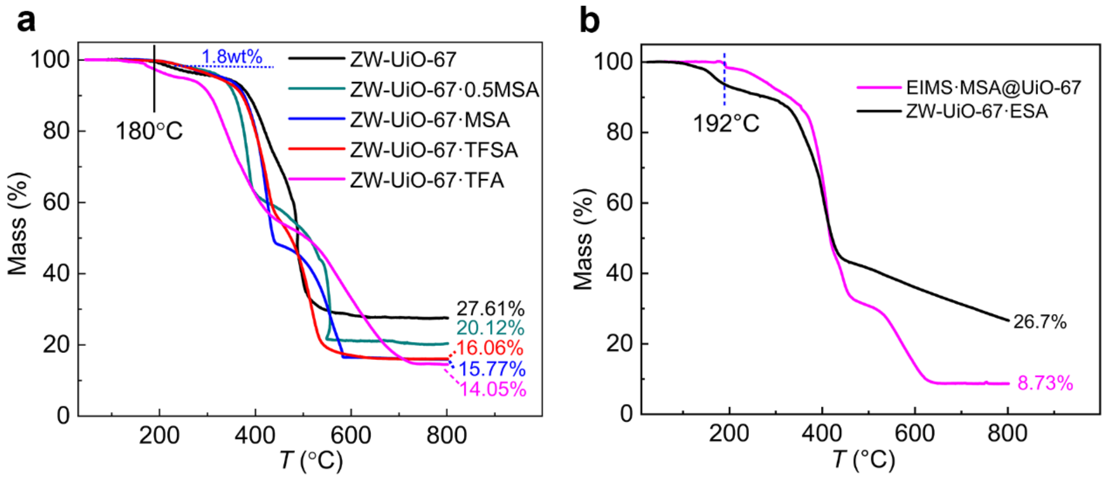


**Supplementary Fig. 16 | Thermogravimetric analysis (TGA) traces**. **a, ZW-UiO-67**, **ZW-UiO-67·HA** and **ZW-UiO-67·0.5MSA** in comparison. The first weight loss occurred at 180 °C for **ZW-UiO-67·MSA**, 200 °C for **ZW-UiO-67·TFSA** and 170 °C for **ZW-UiO-67·TFA**. **b**, Plots of **EIMS·MSA@ZW-UiO-67** and **ZW-UiO-67·ESA**. Remained mass percentages from 8.73 wt% to 27.61 wt% are the final product $ZrO_2$ at 800 °C (see also Supplementary Table 2).

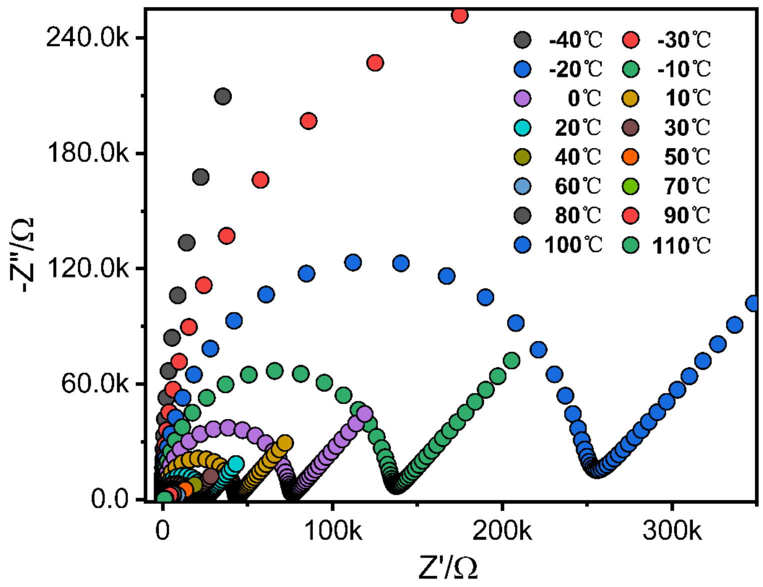


**Supplementary Fig. 17 |** Temperature dependent Nyquist plots of $a_g$ZW-UiO-67·MSA from –40 °C to 110 °C, corresponding to Fig. 2c.

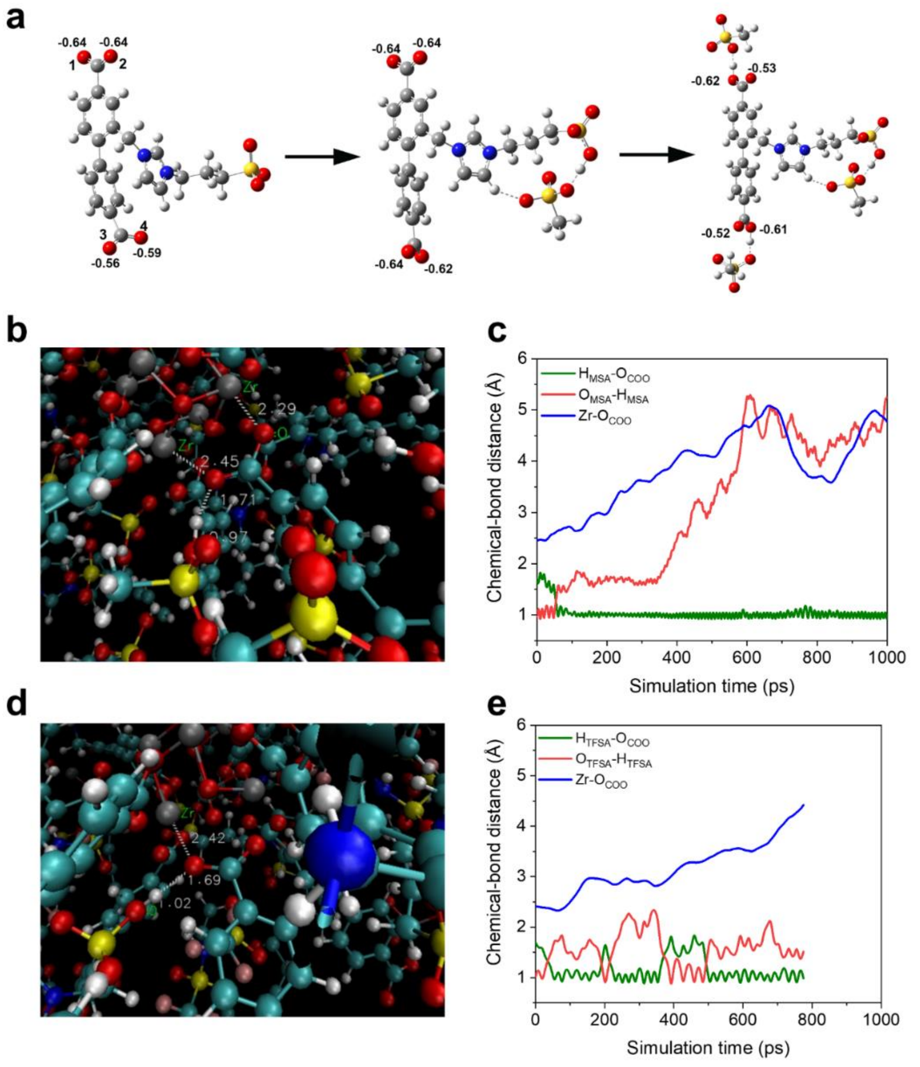


**Supplementary Fig. 18 | Computer calculation suggest for the key role of HA in enabling the meltability of ZW-UiO-67·HA. a**, DFT calculation of interactions between BPDC-MIMS$^{2-}$ and MSA. Optimized structure (left) (via DFT calculation) of BPDC-MIMS$^{2-}$ (deprotonated), and its coordination with one (middle) and more (right) MSA molecules. Carboxylate O atoms (1, 2, 3 and 4) are labeled with negative charges that changed with the MSA coordination. The negative charges of the four carboxylate O atoms decrease with the increasing number of MSA molecules, being consistent with the MSA mass-dependent melting of meltable **ZW-UiO-67·MSA** and unmeltable **ZW-UiO-67·0.5MSA**. **b**, HA (MSA, TFSA) within **ZW-UiO-67·HA** simulated via *Ab initio* MD (AIMD). Local structure of MSA and **ZW-UiO-67** within **ZW-UiO-67·MSA** shown with $H_{MSA}$-$O_{COO}$ interaction and Zr-$O_{COO}$ bonding, and dynamic evolution of chemical-bond distances induced by the hydrogen bond interaction (**c**). The Zr-$O_{COO}$ of 2.29 Å ~ 2.45 Å shows a $\eta^1$-monodentated model (dashed lines) as that indicted by EXAFS (Fig. 3 in main text). **d**, Local structure of TFSA and ZW-UiO-67 within ZW-UiO-67·MSA shown with $H_{TFSA}$-$O_{COO}$ interaction and Zr-$O_{COO}$ bonding, and dynamic evolution of chemical-bond distances induced by the hydrogen bond interaction (**e**). The protonated carboxylate $O_{COO}$ by Brønsted acid HA and the departure of Zr-$O_{COO}$ bond observed by AIMD well agrees with that depressed negative charges on $O_{COO}$ of BPDC-MIMS$^{2-}$ (meaning weakened Zr-$O_{COO}$) upon HA coordination investigated via DFT calculation.

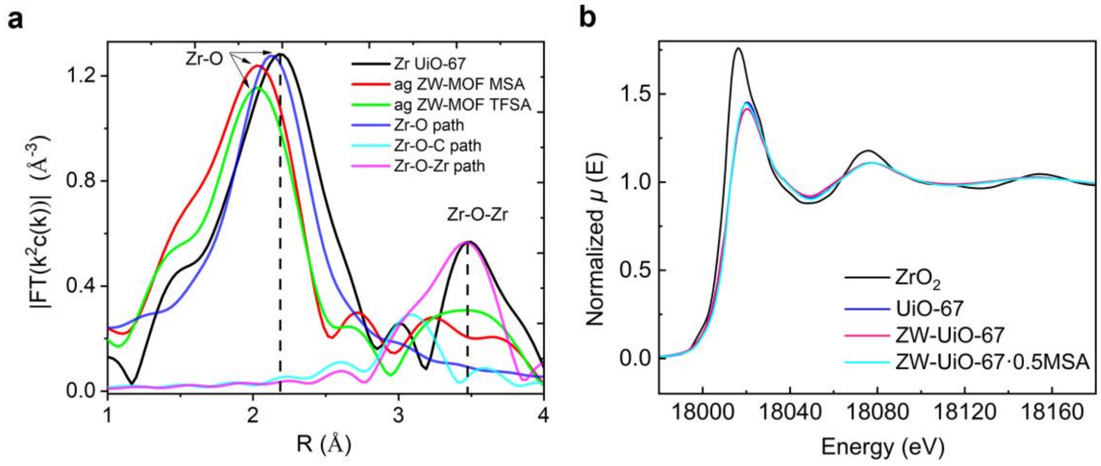


**Supplementary Fig. 19 | XANES and χ(R) space spectra in comparison**. **a**, The strength of different coordination shell path contribution of Zr-O-Zr and Zr-O in χ(R) space spectra in comparison. **b,** XANES μ(E) spectra observed at the K edge of the Zr atoms in UiO-67, $ZrO_2$, **ZW-UiO-67** and **ZW-UiO-67·0.5MSA** at 298 K.

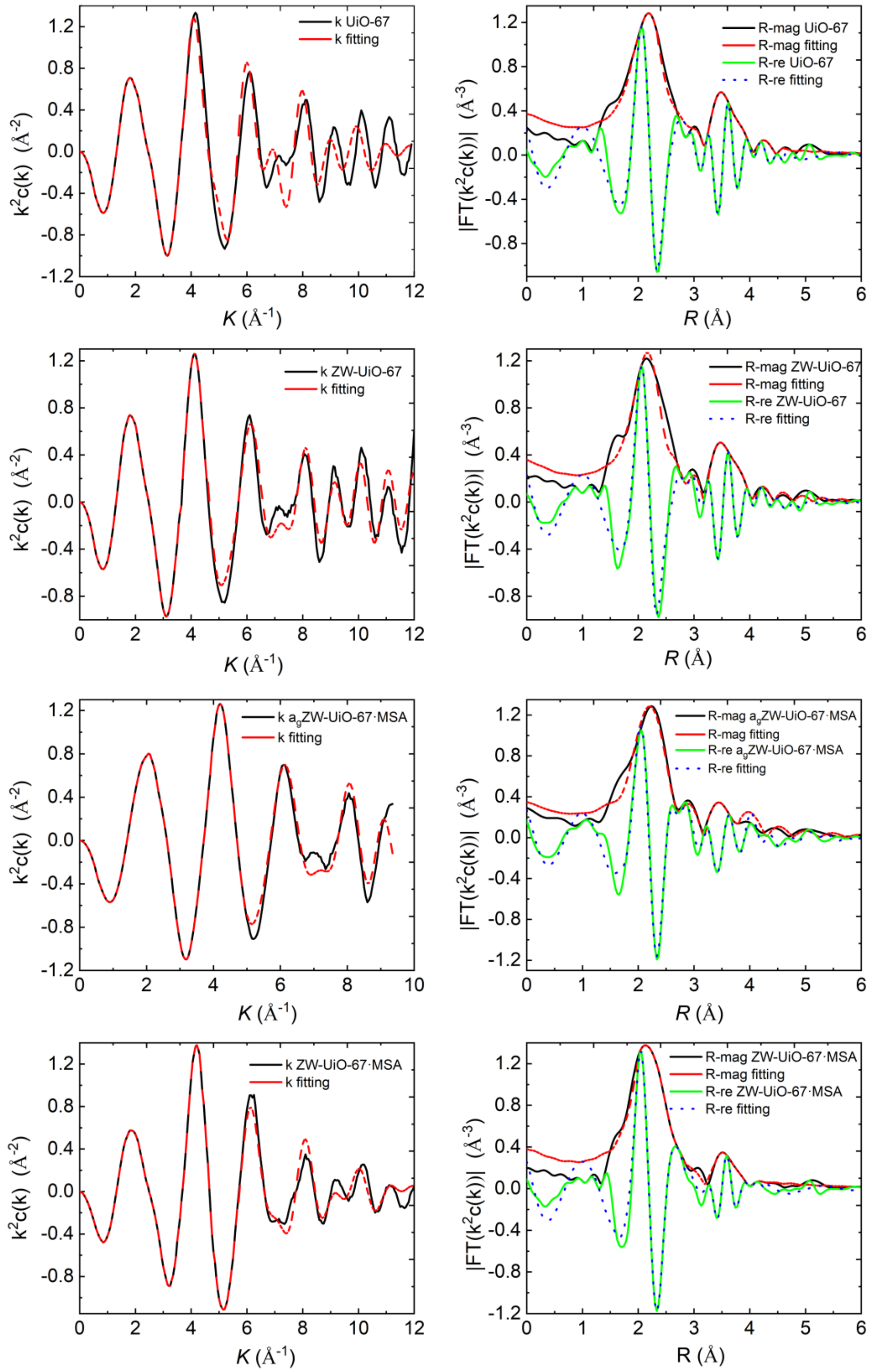
k UiO-67
k fitting
K (Å-1)
k2c(k) (Å-2)
R-mag UiO-67
R-mag fitting
R-re UiO-67
R-re fitting
R (Å)
|FT(k2c(k))| (Å-3)
k ZW-UiO-67
k fitting
R-mag ZW-UiO-67
R-mag fitting
R-re ZW-UiO-67
R-re fitting
k agZW-UiO-67·MSA
k fitting
R-mag agZW-UiO-67·MSA
R-mag fitting
R-re agZW-UiO-67·MSA
R-re fitting
k ZW-UiO-67·MSA
k fitting
R-mag ZW-UiO-67·MSA
R-mag fitting
R-re ZW-UiO-67·MSA
R-re fitting

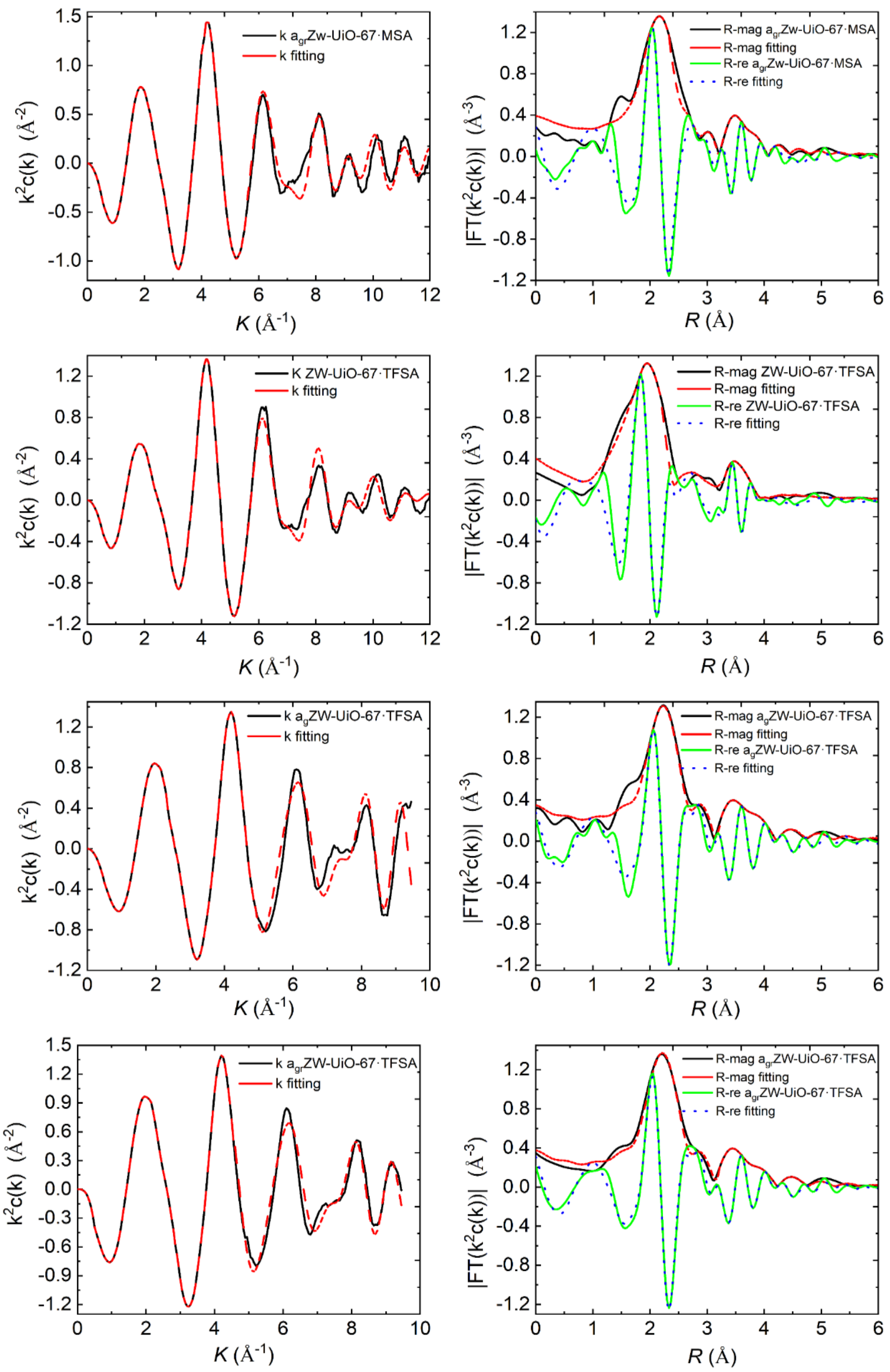


**Supplementary Fig. 20 | K- (left) and R-space (right) EXAFS spectra.** Comparison between experimental and best fit for UiO-67, **ZW-UiO-67**, **ZW-MOF·HA**, **a$_{g}$ZW-UiO-67·HA**, and **a$_{gr}$ZW-UiO-67·HA** (HA = MSA, TFSA), in K- (left) and R-space (right) EXAFS spectrum.

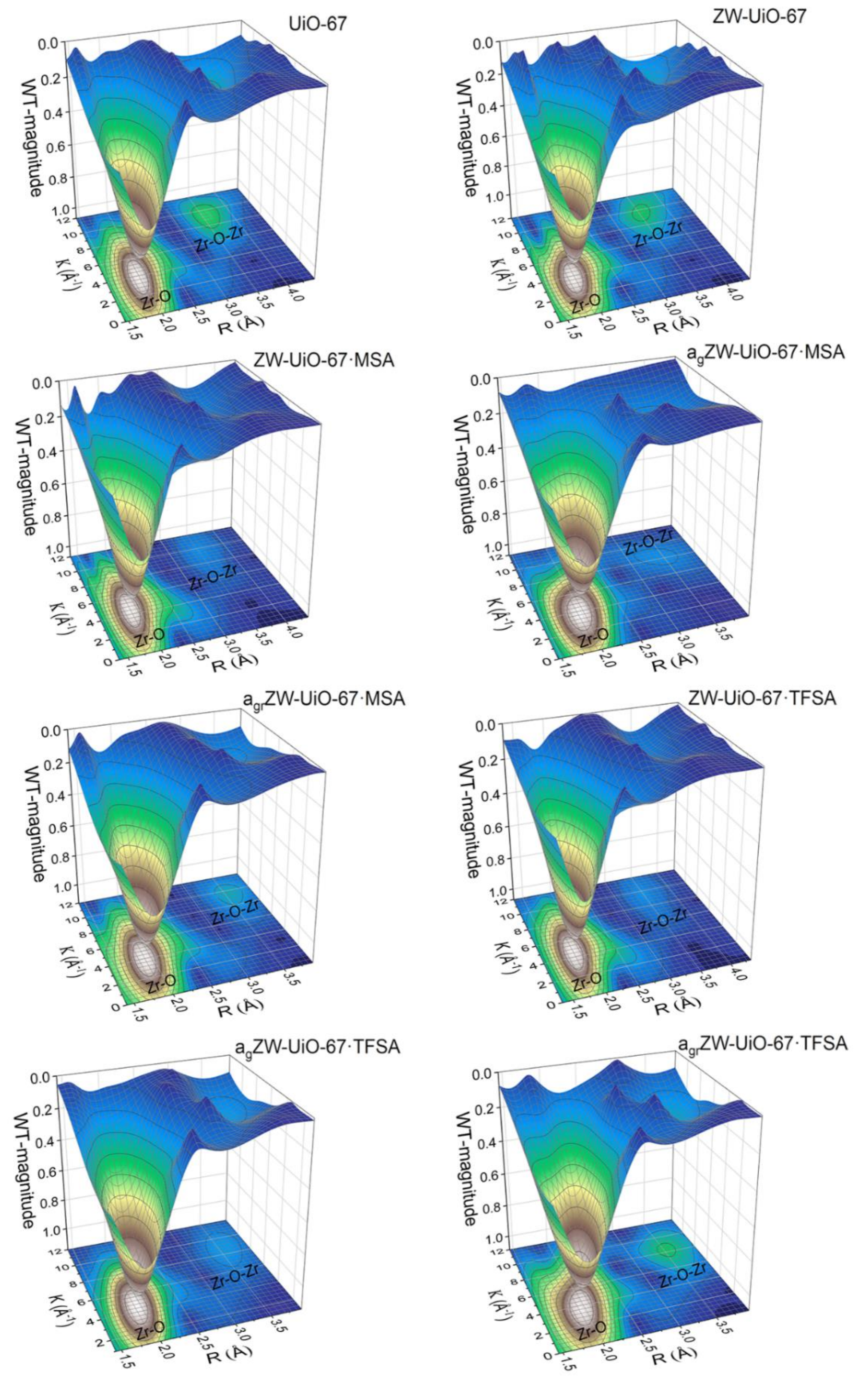


**Supplementary Fig. 21 |** Wavelet transform EXAFS of ZW-UiO-67·MSA, $a_g$ZW-UiO-67·MSA, $a_{gr}$ZW-UiO-67·MSA, ZW-UiO-67·TFSA, $a_g$ZW-UiO-67·TFSA, $a_{gr}$ZW-UiO-67·TFSA, ZW-UiO-67 and parent UiO-67.

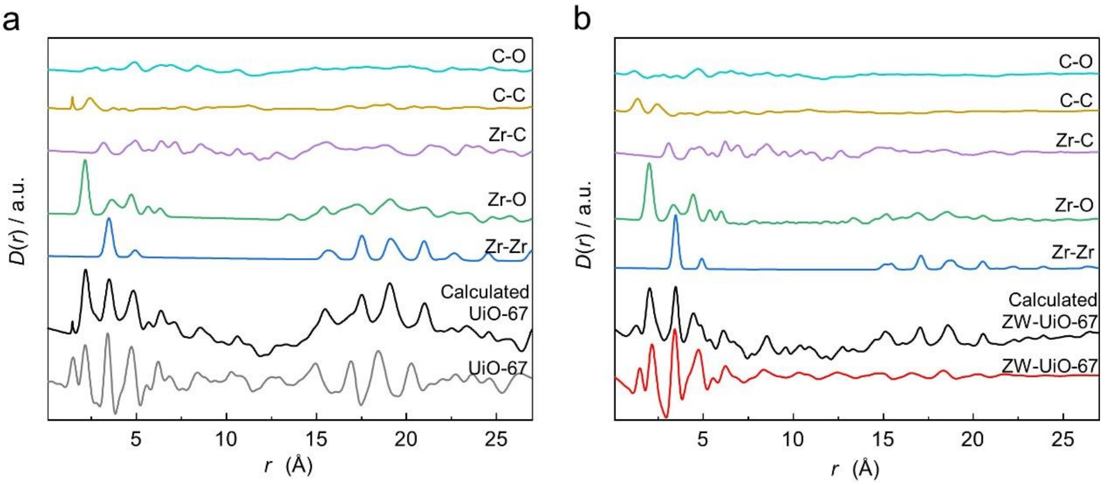


**Supplementary Fig. 22 |** Partial pair distribution functions and X-ray total pair distribution function for UiO-67 (**a**) and ZW-UiO-67 (**b**) calculated from their crystal structures using PDFGui, and experimental PDF data (lowermost plots-gray and red lines). Detailed atom-to-atom distances obtained from main peaks of PDF plots for **a$_g$ZW-UiO-67·HA** are shown in Supplementary Table 4 in comparison.

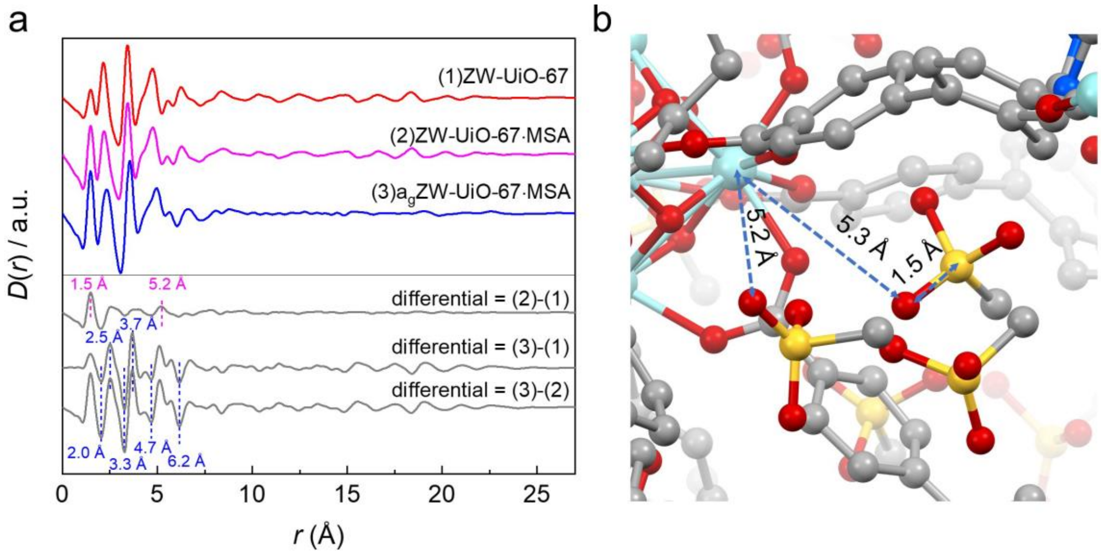


**Supplementary Fig. 23 | Compared differentials of PDF**. **a**, Differentials (gray) between PDF of **ZW-UiO-67** and **ZW-UiO-67·MSA** and **a$_g$ZW-UiO-67·MSA**. **b**, Local molecular dynamics simulated structure of **ZW-UiO-67·MSA** at 298K. Bond lengths and atom to atom distances related to **a** panel are labeled and marked as dashed lines.

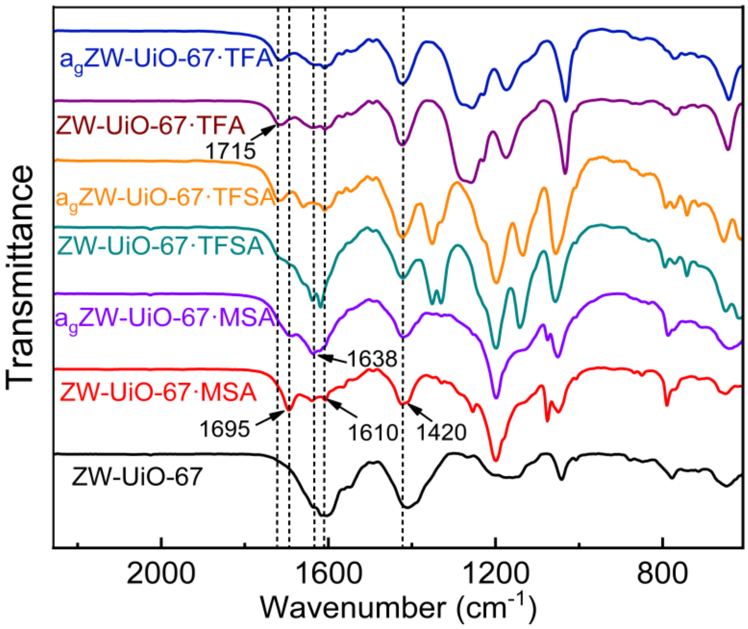


**Supplementary Fig. 24 | Comparison of solid FT-IR spectra at room temperature**. Solid FT-IR spectra of **ZW-UiO-67**, **ZW-UiO-67·HA** and **a$_g$ZW-UiO-67·HA** from 700 cm$^{-1}$ to 2300 cm$^{-1}$ range for clarity and comparison. Bands of 1610 cm$^{-1}$ and 1638 cm$^{-1}$ are assigned to $\nu_a$(COO), while 1420 cm$^{-1}$ belongs to $\nu_s$(COO) stretching vibration. Bands 1715 cm$^{-1}$ and 1695 cm$^{-1}$ may be attributed to the shifted $\nu_a$(COO) of protonated carboxylate.

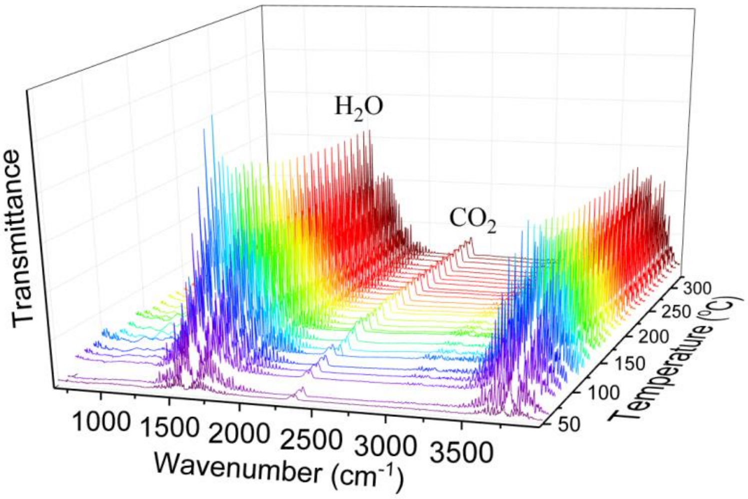


**Supplementary Fig. 25 | Online gas-phase FT-IR spectra with temperature increasing**. In-situ gas-phase FT-IR of **ZW-UiO-67·MSA** measured from 25 °C to 320 °C, with the dominant stretching vibration bands of $H_2O$ (1250 cm$^{-1}$-2000 cm$^{-1}$, 3400 cm$^{-1}$-4000 cm$^{-1}$) and $CO_2$ (~2200 cm$^{-1}$).

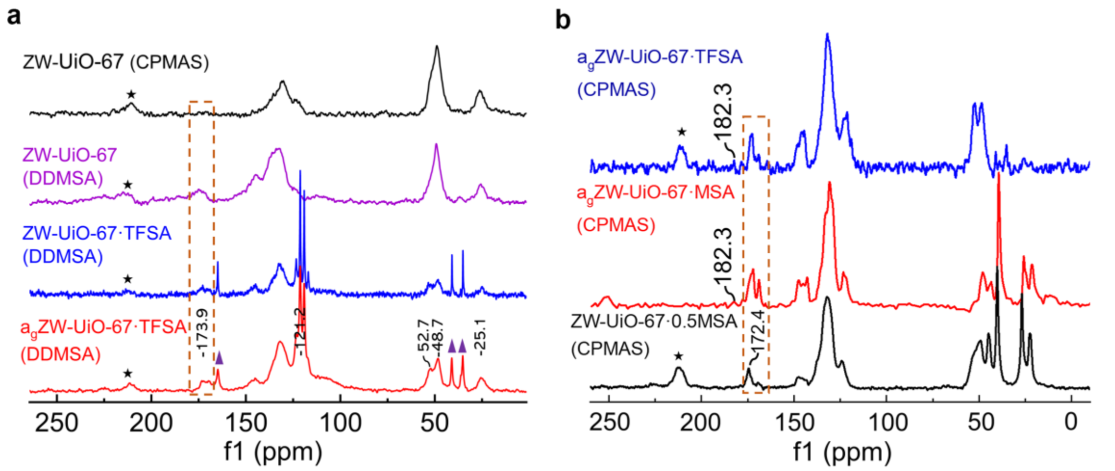


**Supplementary Fig. 26 | Solid-state $^{13}$C NMR spectra at room temperature. a**, Solid-state $^{13}$C NMR spectra of CP MAS (at 12 kHz) of **ZW-UiO-67**, and DD MAS (at 12 kHz) of **ZW-UiO-67**, **ZW-UiO-67·TFSA** and **a$_g$ZW-UiO-67·TFSA**. Enclosed area indicates the missed CP MAS resonance of $C_{COO}$ in **ZW-UiO-67** and the appeared DD MAS signal in comparison. Spinning sideband are marked with asterisks. Signals signed with triangle are $^{13}$C resonances of DMF (*N,N*-dimethylformamide) solvent. 121.2 ppm is the resonance signal of $-CF_3$ in TFSA (*N,N*-bis(trifluoromethanesulfonyl) amide). **b**, Appeared $C_{COO}$ resonance in CP MAS of $^{13}$C NMR spectra of **a$_g$ZW-UiO-67·MSA** and **a$_g$ZW-UiO-67·TFSA** and **ZW-UiO-67·0.5MSA** due to protonated $C_{COO}$. Enclosed rectangle indicates the appeared $C_{COO}$ resonance, as compared to that missed signal of **ZW-UiO-67** (See also Fig. 4e).

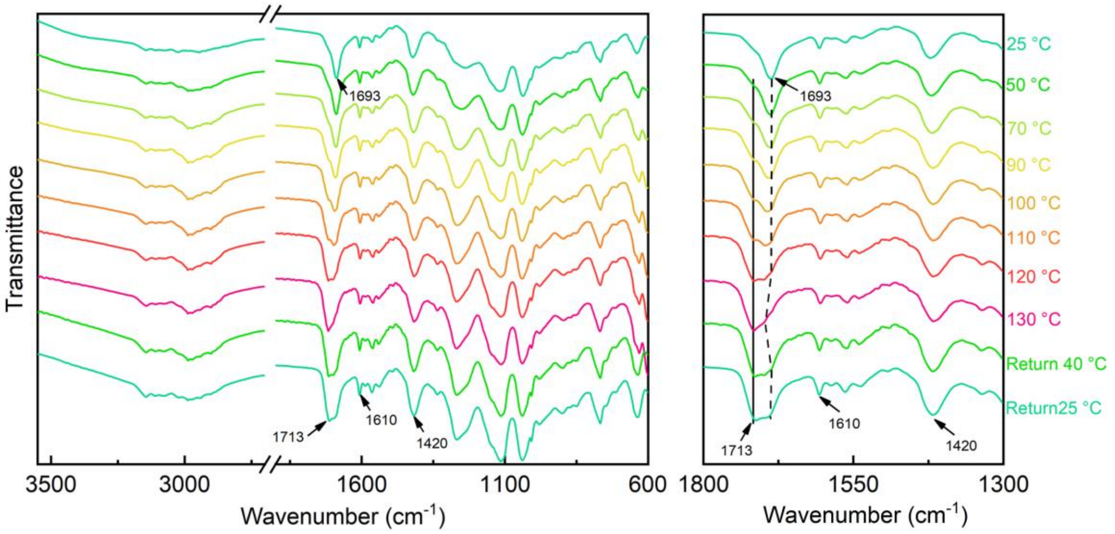


**Supplementary Fig. 27 | Variable temperature in-situ FT-IR of ZW-UiO-67·MSA**. The right panel of the spectra from 1300 cm$^{-1}$ to 1800 cm$^{-1}$ range, with the guiding eye lines to show the change of the carboxylate C=O band.

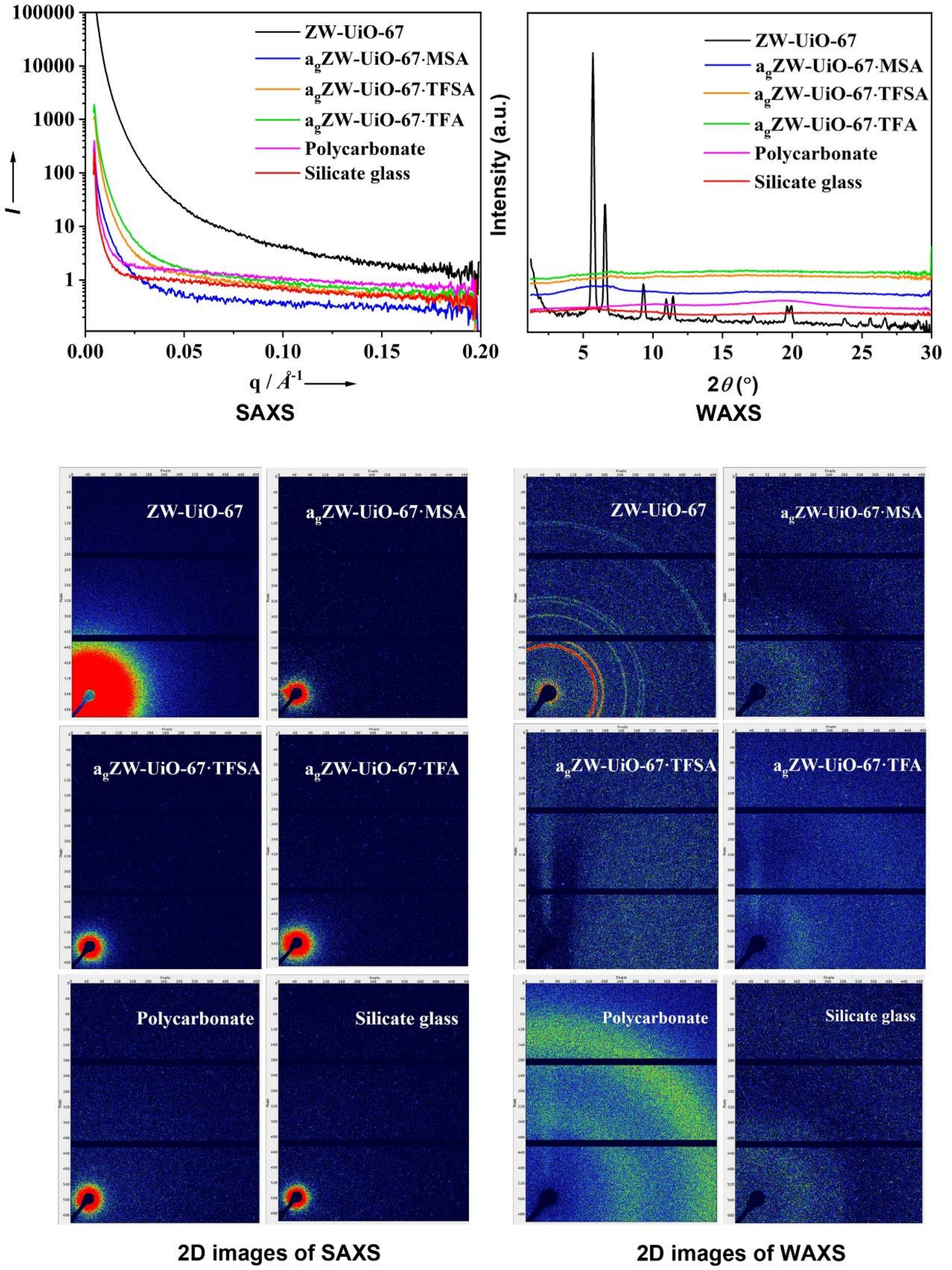


**Supplementary Fig. 28 | SAXS (small-angle X-ray scattering) and WAXS (wide-angle X-ray scattering)**. Top: SAXS and PXRD patterns of WAXS of crystalline **ZW-UiO-67**, amorphous **a$_g$ZW-UiO-67·HA**, polycarbonate and silicate glass. Bottom: 2D images of SAXS and WAXS in comparison.

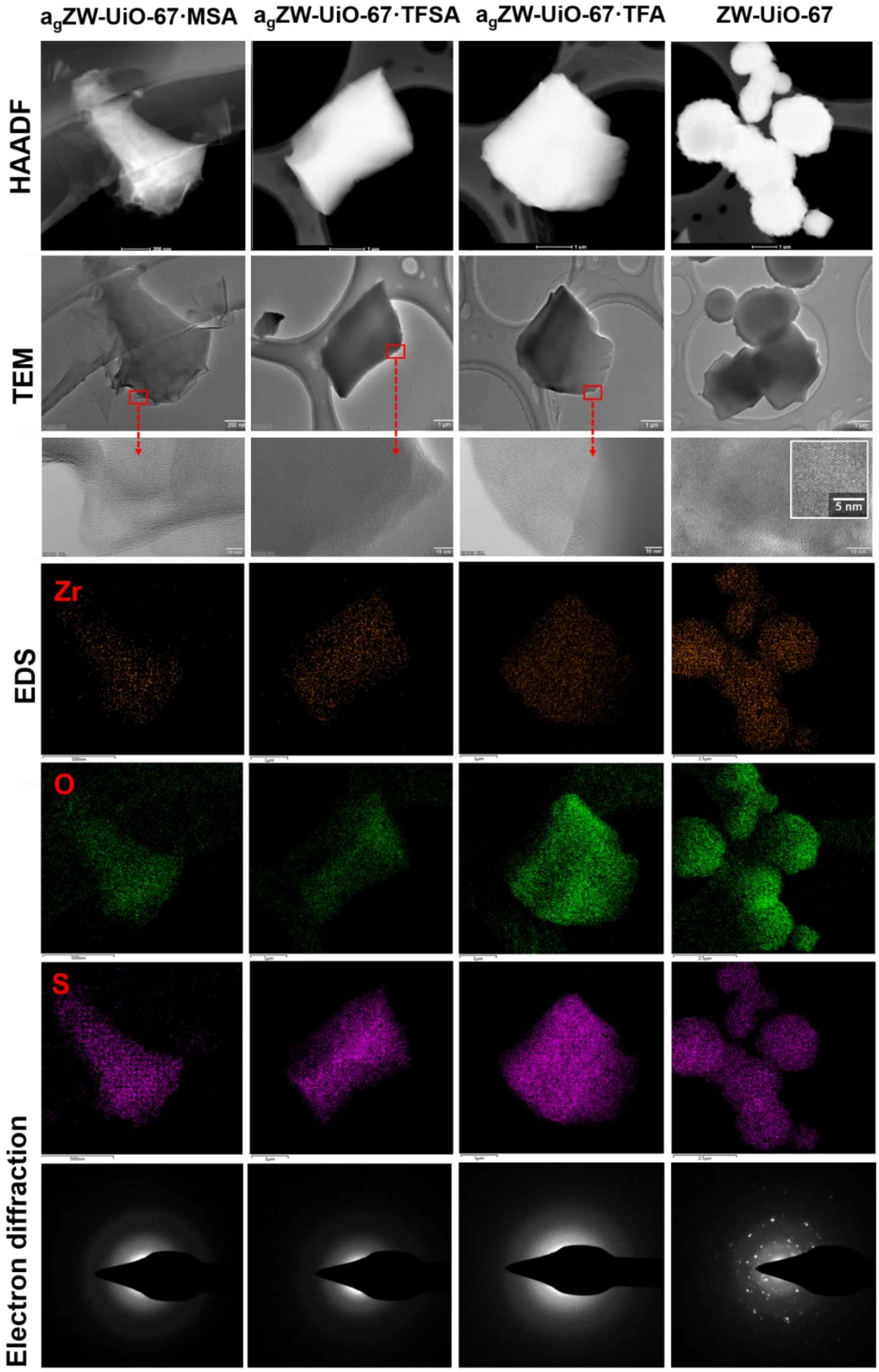


**Supplementary Fig. 29 |** Transmission electron microscopy images, Energy-dispersive X-ray spectroscopy (EDX) analysis with elemental maps, electron diffraction of glassy **$a_g$ZW-UiO-67·HA** and crystalline **ZW-UiO-67** samples. HAADF, high-angle annular dark-field.

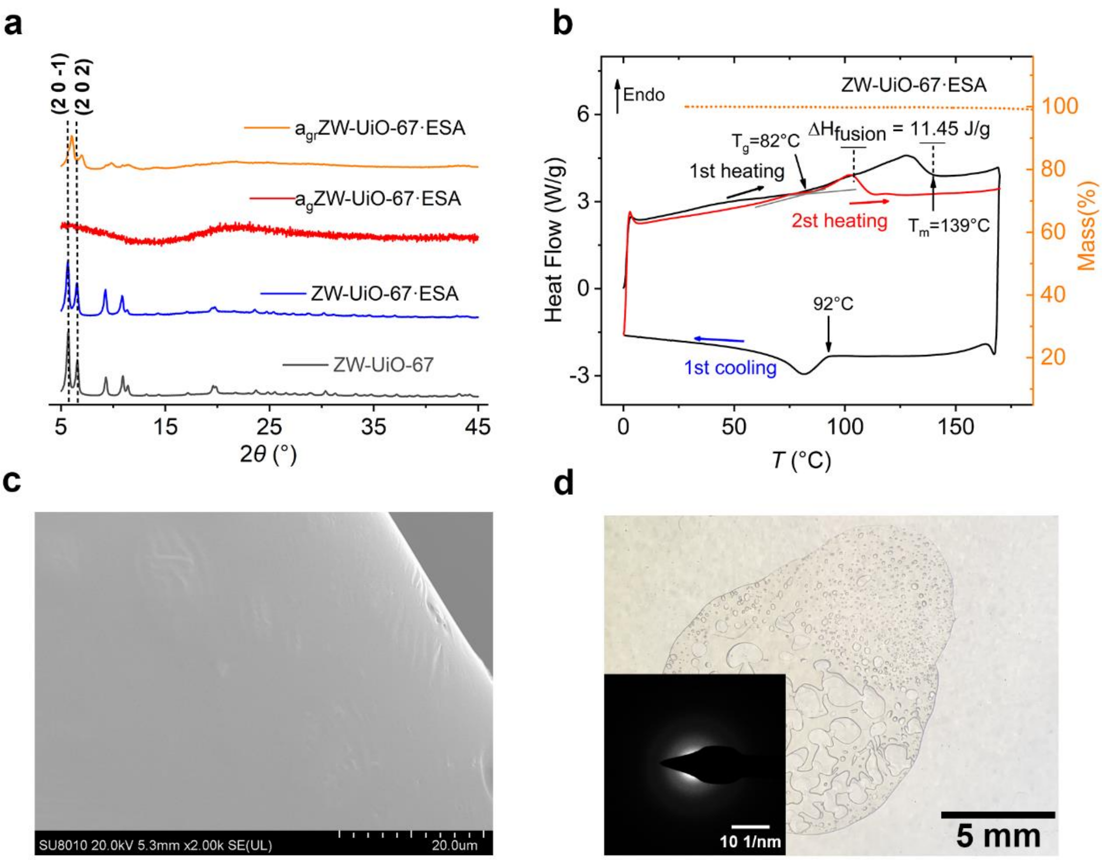


**Supplementary Fig. 30 | Characteristics of ZW-UiO-67·ESA and derivates.** **a**, PXRD patterns of crystalline **ZW-UiO-67** and **ZW-UiO-67·ESA**, glassy **$a_g$ZW-UiO-67·ESA** and **$a_{gr}$ZW-UiO-67·ESA** in comparison. **b**, Cyclic DSC curve of **ZW-UiO-67·ESA** within 0~170 °C, with yellow TGA trace, melting temperature ($T_m$), glass transition temperature ($T_g$) and fusion enthalpy indicated. The exothermal peak (92 °C) may be phase transition reminiscent of **ZW-UiO-67·MSA**. **c**, SEM image of **$a_g$ZW-UiO-67·ESA**. **d**, Photography of transparent glass **$a_g$ZW-UiO-67·ESA** quenched from 140 °C (inset: electron diffraction pattern from glass).

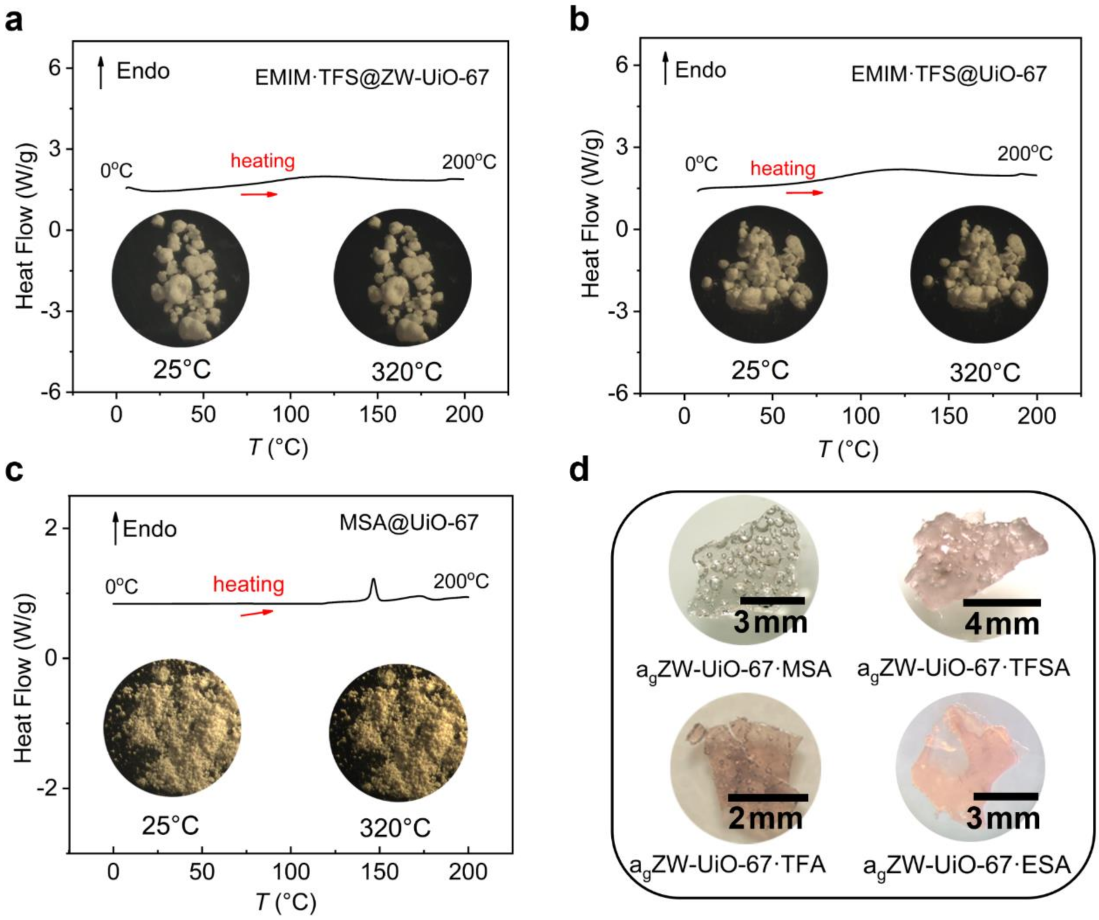


**Supplementary Fig. 31 | Selected DSC curves (0-200 °C) and in-situ photography of heated (320 °C) composites derived from ZW-UiO-67 and UiO-67 in comparison. a**, **EMIM·TFS@ZW-UiO-67**. **b**, **EMIM·TFS@UiO-67**. **c**, **MSA@UiO-67**. The endothermal event at 152 °C of **MSA@UiO-67** may be ascribed to the expelled MSA upon heat. **d**, Microscopic photography of melt-quenched glass **$a_g$ZW-UiO-67·HA** in comparison. See also the Supplementary Table 2,5 of components and more information for ILs (EMIM·TFS etc.) and nanocomposites in this study.

## Supplementary Section 2. Characterization related to ZW-DUT-5 and its derivatives.

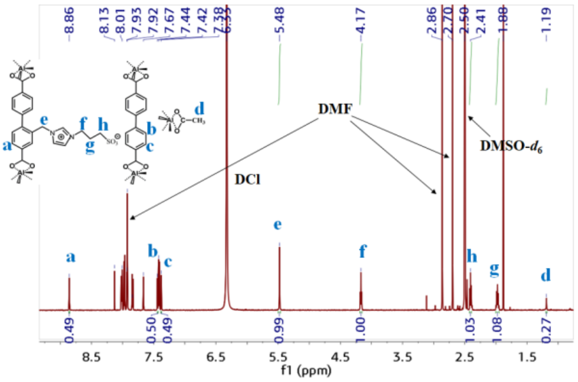


**Supplementary Fig. 32 | $^{1}$H NMR spectra of the mixed-linker ZW-DUT-5 with a stoichiometric formula $Al(OH)(BPDC\text{-}MIMS)_{0.72}(BPDC)_{0.18}(O_2CCH_3)_{0.13}$.**

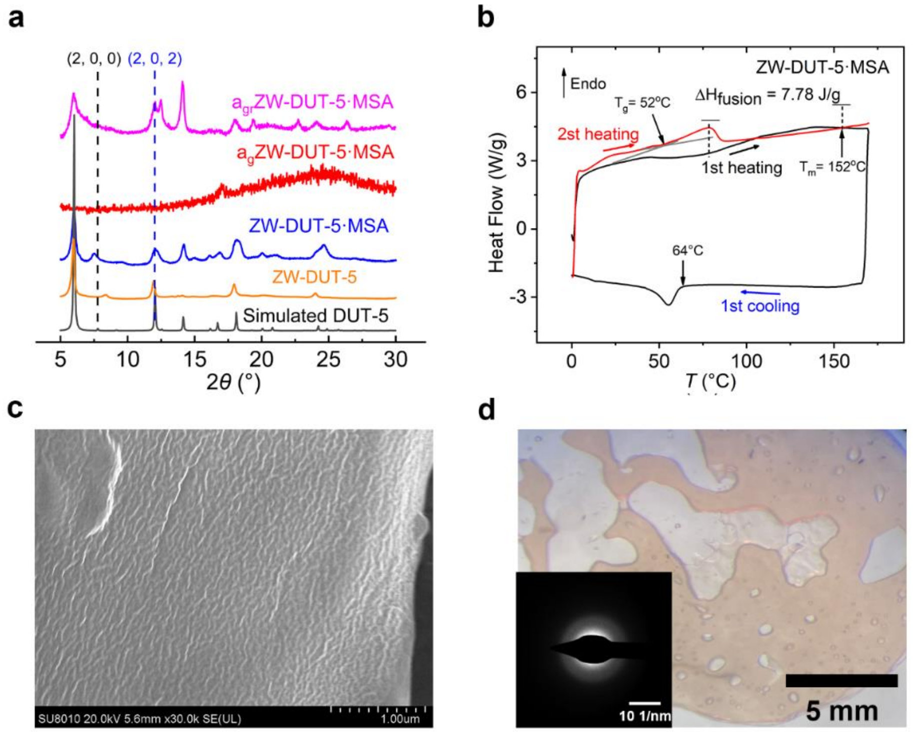


**Supplementary Fig. 33 | Characteristics of ZW-DUT-5·MSA and derivates. a**, PXRD patterns for **ZW-DUT-5**, **ZW-DUT-5·MSA**, **$a_g$ZW-DUT-5·MSA** and **$a_{gr}$ZW-DUT-5·MSA** in comparison. Simulation pattern of DUT-5 is obtained from the crystal data reported[4]. **b**, Cyclic DSC curve of **ZW-DUT-5·MSA** (0 °C ~170 °C). The exothermal event at 64 °C is reminiscent of that phase transition of **ZW-UiO-67·MSA** and **ZW-UiO-67·ESA**. **c**, SEM image, and **d**, Photography of transparent glass **$a_g$ZW-DUT-5·MSA** at room temperature (inset: electron diffraction pattern from glass **$a_g$ZW-DUT-5·MSA**). Here $T_g$ (65 °C) is lower than that of **ZW-UiO-67·ESA** (82 °C), **ZW-UiO-67·MSA** (104 °C), **ZW-UiO-67·TFSA** (104 °C) and **ZW-UiO-67·TFA** (105 °C). Measured melting enthalpy $\Delta H_{fusion}$ = 7.82 J/g lies within the 2.12-11.45 J/g range of **ZW-UiO-67·HA** (Supplementary Table 6).

## Supplementary Section 3. Characterization related to ZW-UiO-68 and its derivatives.

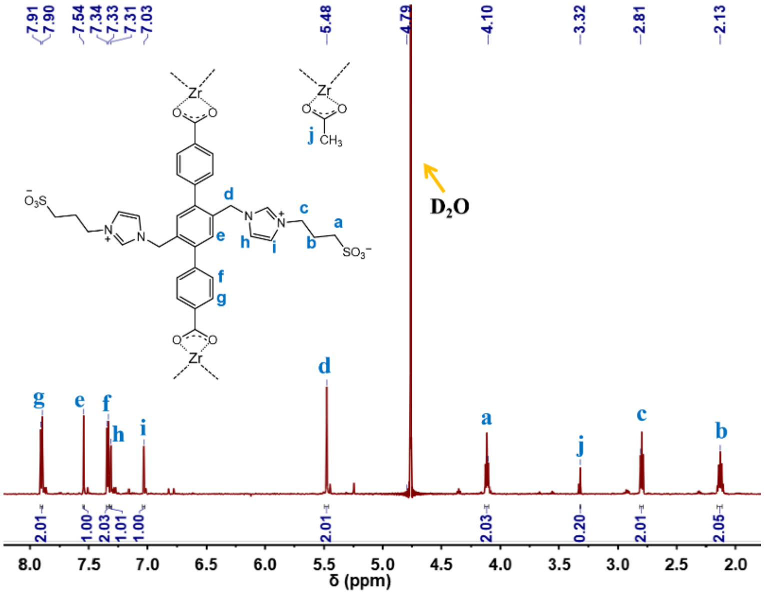

**Supplementary Fig. 34 | $^{1}$H NMR spectra of ZW-UiO-68 digested in NaOH solution of $D_2O$.** Rationalized formula of **ZW-UiO-68**: $Zr_6O_4(OH)_4(TPDC\text{-}MIMS)_{5.62}(CH_3CO_2)_{0.76}$. Inset shows the bis-substituted ligand $H_2$TPDC-MIMS with zwitterion group EIMS (1-ethylimidazolium-3-propanesulfonate).

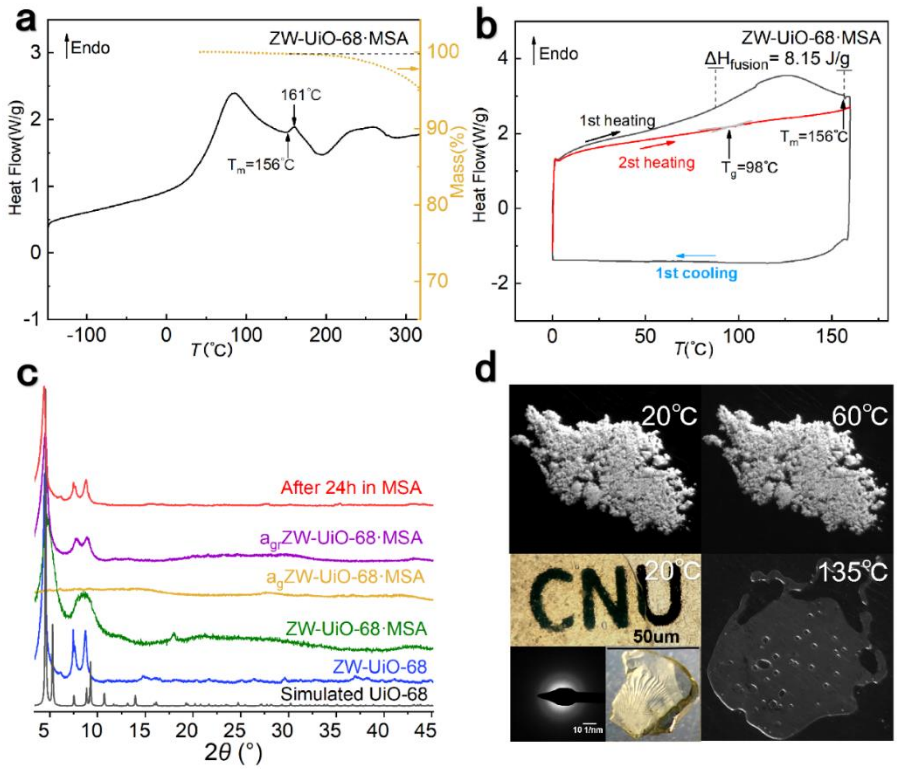


**Supplementary Fig. 35 | Characteristics of ZW-UiO-68·MSA and derivates**. **a**, DSC plot from -150 °C to 320 °C, and TGA trace from 25 °C to 320 °C of **ZW-UiO-68·MSA**, showing no weight loss before the offset $T_m$ = 156 °C. **b**, Cyclic curve of DSC within 0 °C–160 °C range. No any peak was recorded during the cooling, indicating no phase transition occurred. **c**, PXRD patterns in comparison of **ZW-UiO-68**, **ZW-UiO-68·MSA**, **a$_g$ZW-UiO-68·MSA**, **a$_{gr}$ZW-UiO-68·MSA** and that of simulated UiO-68. **d**, In-situ morphology photography of powdery **ZW-UiO-68·MSA** at room temperature 20 °C and 60 °C, liquid at 135 °C and the melt-quenched transparent glass at 20 °C (bottom inset: electron diffraction, and optical micrography of fracture pattern of bulk glass. Scale bar: 50 μm).

## Supplementary Section 4. Supplementary Tables.

**Supplementary Table 1 |** BET, pore volumes and pore sizes of **ZW-UiO-67, ZW-UiO-67·0.5MSA, ZW-MOF·HA, $a_g$ZW-MOF·HA, $a_{gr}$ZW-MOF·HA, EIMS·MSA @UiO-67** and **UiO-67**.

| Composites | $N_2$ adsorption data | | |
|---|---|---|---|
| | BET ($m^2/g$) | pore volume ($cm^3/g$) | pore size (Å) |
| **ZW-UiO-67** | 300.23 | 0.426 | ~6/~12 |
| **ZW-UiO-67·MSA** | 12.92 | 0.033 | ignorable |
| **ZW-UiO-67·0.5MSA** | 68.14 | 0.295 | ~14 |
| **ZW-UiO-67·TFSA** | 12.11 | 0.029 | ignorable |
| **$a_g$ZW-UiO-67·MSA** | 0.23 | 0.027 | ignorable |
| **$a_g$ZW-UiO-67·TFSA** | 0.33 | 0.028 | ignorable |
| **$a_g$ZW-UiO-67·TFA** | 1.48 | 0.028 | ignorable |
| **$a_{gr}$ZW-UiO-67·MSA**[a] | 256.46 | 0.155 | ~6/~12 |
| **$a_{gr}$ZW-UiO-67·TFSA**[b] | 29.00 | 0.131 | ~15 |
| **$a_{gr}$ZW-UiO-67·TFA**[c] | 13.4 | 0.055 | ignorable |
| **EIMS·MSA @UiO-67** | 11.80 | 0.028 | Ignorable |
| **UiO-67** | 2432 | 0.954 | ~12 |

[a]Sample of **$a_{gr}$ZW-UiO-67·MSA** was produced by 3 h refluxing in methanol. [b,c]**$a_{gr}$ZW-UiO-67·TFSA/TFA** were obtained via 3-minute soaking in methanol with stirring at room temperature.

**Supplementary Table 2 |** Compositions of **ZW-UiO-67, ZW-UiO-67·0.5MSA, ZW-UiO-67·HA, a$_{gr}$ZW-UiO-67·MSA,** and **EIMS·MSA@UiO-67.** HA = MSA, TFSA, TFA, ESA.

| | ZW-UiO-67 (mg) | HA (μl) | Stoichiometry Formula[a] (L = BPDC-MIMS) | Formula weight | $ZrO_2$ ($_{wt}$%)[b] Calc./meas. |
|---|---|---|---|---|---|
| **ZW-UiO-67** | / | / | $Zr_6O_4(OH)_4(L)_{5.4}(O_2CCH_3)_{1.2}$ | ~3124.8 | ~23.7/27.6 |
| **ZW-UiO-67·MSA** | 80 | 34 | $Zr_6O_4(OH)_4(L)_{5.4}\cdot(O_2CCH_3)_{1.2}\cdot(MSA)_{20.5}$ | ~5092.8 | ~14.5/15.7 |
| **ZW-UiO-67·0.5MSA** | 80 | 17 | $Zr_6O_4(OH)_4(L)_{5.4}\cdot(O_2CCH_3)_{1.2}\cdot(MSA)_{10.2}$ | ~4104.0 | ~18.0/20.1 |
| **ZW-UiO-67·TFSA** | 80 | 34 | $Zr_6O_4(OH)_4(L)_{5.4}\cdot(O_2CCH_3)_{1.2}\cdot(HTFSA)_{6.4}$ | ~4923.2 | ~15.0/16.0 |
| **ZW-UiO-67·TFA** | 80 | 34 | $Zr_6O_4(OH)_4(L)_{5.4}\cdot(O_2CCH_3)_{1.2}\cdot(TFA)_{15.0}$ | ~5374.8 | ~13.8/14.1 |
| **ZW-UiO-67·ESA** | 80 | 34 | $Zr_6O_4(OH)_4(L)_{5.4}\cdot(O_2CCH_3)_{1.2}\cdot(ESA)_{16.3}$ | ~4916.6 | ~15.0/26 |
| **EIMS·MSA@UiO-67** | UiO-67 (80 mg) & ILs (81 μL) | | $Zr_6O_4(OH)_4(BPDC)_{5.4}\cdot(O_2CCH_3)_{1.2}\cdot[EIMS]_{4.93}[MSA]_{18.7}$ | ~4935.7 | ~15.0/8.73 |
| **a$_{gr}$ZW-UiO-67·MSA** | / | / | $Zr_6O_4(OH)_4(L)_{5.4}\cdot(O_2CCH_3)_{1.2}\cdot(MSA)_{12.3}$ | ~4305.6 | Unmeasured |

| HA or ILs used in this study | | | | | | |
|---|---|---|---|---|---|---|
| **HA/ILs**[c] | EIMS·MSA | EMIM·TFA | MSA | TFSA[d] | TFA | ESA |
| ρ (g/ml) | 1.42 | 1.38 | 1.48 | 1.892 | 1.696 | 1.35 |

[a]Stoichiometry formula is based on **ZW-UiO-67** which was determined through $^1$H NMR (Supplementary Fig. 2), and volume of HA or ionic liquid equals to the pore of **ZW-UiO-67** used. [b] Data is obtained from the TGA traces (Supplementary Fig.16). [c] Ionic liquids (ILs) were synthesized as that illustrated in Supplementary Fig. 1. [d] Melt point of TFSA (bis(trifluoromethane)sulfonimide) is 52 °C, here used as a liquid under 55 °C.

**Supplementary Table 3 |** Structural parameters extracted from the K-edge Pd $\chi$(R) space spectra fitting of **UiO-67, ZW-UiO-67, $a_g$ZW-UiO-67·HA** and **$a_{gr}$ZW-UiO-67·HA.** HA = MSA, TFSA.

| Sample | | Reduced Chi-square ($\chi_v^2$) | | R-factor（%） |
|---|---|---|---|---|
| **UiO-67** | | 892.04 | | 0.0402 |
| amp/ $S_0^2$ | $N_{(Zr\text{-}O\ path)}$ | $R_{(Zr\text{-}O\ path)}$ (Å) | $\sigma^2_{(Zr\text{-}O\ path)}$ ($10^{-3}$Å$^2$) | $\Delta E_0$ (eV) |
| 1.03+/- 0.14 | 8 | 2.173 ± 0.038 | 3.3+/-1.6 | -3.21+/- 1.67 |
| amp/ $S_0^2$ | $N_{(Zr\text{-}Zr\ path)}$ | $R_{(Zr\text{-}Zr\ path)}$ (Å) | $\sigma^2_{(Zr\text{-}Zr\ path)}$ ($10^{-3}$Å$^2$) | $\Delta E_0$ (eV) |
| 0.82+/- 0.14 | 4 | 3.558 ± 0.062 | 4.0+/-1.8 | 3.71+/-1.91 |

| Sample | | Reduced Chi-square ($\chi_v^2$) | | R-factor（%） |
|---|---|---|---|---|
| **ZW-UiO-67** | | 684.89 | | 0.0463 |
| amp/ $S_0^2$ | $N_{(Zr\text{-}O\ path)}$ | $R_{(Zr\text{-}O\ path)}$ (Å) | $\sigma^2_{(Zr\text{-}O\ path)}$ ($10^{-3}$Å$^2$) | $\Delta E_0$ (eV) |
| 1.03 | 7.85 ± 1.67 | 2.189 ± 0.054 | 3.4+/-1.1 | 2.12+/- 1.49 |
| amp/ $S_0^2$ | $N_{(Zr\text{-}Zr\ path)}$ | $R_{(Zr\text{-}Zr\ path)}$ (Å) | $\sigma^2_{(Zr\text{-}Zr\ path)}$ ($10^{-3}$Å$^2$) | $\Delta E_0$ (eV) |
| 0.82 | 3.82 ± 1.42 | 3.584 ± 0.088 | 4.0+/-1.7 | 4.68+/-2.15 |

| Sample | | Reduced Chi-square ($\chi_v^2$) | | R-factor（%） |
|---|---|---|---|---|
| **ZW-UiO-67·MSA** | | 644.38 | | 0.0482 |
| amp/ $S_0^2$ | $N_{(Zr\text{-}O\ path)}$ | $R_{(Zr\text{-}O\ path)}$ (Å) | $\sigma^2_{(Zr\text{-}O\ path)}$ ($10^{-3}$Å$^2$) | $\Delta E_0$ (eV) |
| 1.03 | 7.82 ±1.14 | 2.176 ± 0.042 | 2.8+/-0.9 | 2.08+/- 1.31 |
| amp/ $S_0^2$ | $N_{(Zr\text{-}Zr\ path)}$ | $R_{(Zr\text{-}Zr\ path)}$ (Å) | $\sigma^2_{(Zr\text{-}Zr\ path)}$ ($10^{-3}$Å$^2$) | $\Delta E_0$ (eV) |
| 0.82 | 3.18 ± 1.07 | 3.562 ± 0.066 | 3.7+/-1.1 | 4.01+/-1.93 |

| Sample | | Reduced Chi-square ($\chi_v^2$) | | R-factor（%） |
|---|---|---|---|---|
| **$a_g$ZW-UiO-67·MSA** | | 197.73 | | 0.0296 |
| amp/ $S_0^2$ | $N_{(Zr\text{-}O\ path)}$ | $R_{(Zr\text{-}O\ path)}$ (Å) | $\sigma^2_{(Zr\text{-}O\ path)}$ ($10^{-3}$Å$^2$) | $\Delta E_0$ (eV) |
| 1.03 | 7.01 ± 1.13 | 2.206 ± 0.071 | 2.9+/-0.9 | 1.87+/- 1.09 |
| amp/ $S_0^2$ | $N_{(Zr\text{-}Zr\ path)}$ | $R_{(Zr\text{-}Zr\ path)}$ (Å) | $\sigma^2_{(Zr\text{-}Zr\ path)}$ ($10^{-3}$Å$^2$) | $\Delta E_0$ (eV) |
| 0.82 | 2.95 ± 0.98 | 3.611 ± 0.089 | 3.7+/-1.5 | 4.07+/-1.88 |

| Sample | Reduced Chi-square ($\chi_v^2$) | R-factor（%） |
|---|---|---|
| **$a_{gr}$ZW-UiO-67·MSA** | 555.40 | 0.0441 |

| amp/ $S_0^2$ | $N_{(Zr\text{-}O\ path)}$ | $R_{(Zr\text{-}O\ path)}$ (Å) | $\sigma^2_{(Zr\text{-}O\ path)}$ ($10^{-3}Å^2$) | $\Delta E_0$ (eV) |
|---|---|---|---|---|
| 1.03 | 7.72 ± 1.38 | 2.189 ± 0.063 | 3.6+/-1.3 | 2.18+/- 1.21 |
| amp/ $S_0^2$ | $N_{(Zr\text{-}Zr\ path)}$ | $R_{(Zr\text{-}Zr\ path)}$ (Å) | $\sigma^2_{(Zr\text{-}Zr\ path)}$ ($10^{-3}Å^2$) | $\Delta E_0$ (eV) |
| 0.82 | 3.18 ± 1.12 | 3.584 ± 0.092 | 4.8+/-1.9 | 6.37+/-3.21 |

| Sample | Reduced Chi-square ($\chi_v^2$) | R-factor（%） |
|---|---|---|
| **ZW-UiO-67·TFSA** | 578.54 | 0.0475 |

| amp/ $S_0^2$ | $N_{(Zr\text{-}O\ path)}$ | $R_{(Zr\text{-}O\ path)}$ (Å) | $\sigma^2_{(Zr\text{-}O\ path)}$ ($10^{-3}Å^2$) | $\Delta E_0$ (eV) |
|---|---|---|---|---|
| 1.03 | 7.84 ± 1.28 | 2.177 ± 0.041 | 2.5+/-1.1 | 1.89+/- 0.98 |
| amp/ $S_0^2$ | $N_{(Zr\text{-}Zr\ path)}$ | $R_{(Zr\text{-}Zr\ path)}$ (Å) | $\sigma^2_{(Zr\text{-}Zr\ path)}$ ($10^{-3}Å^2$) | $\Delta E_0$ (eV) |
| 0.82 | 3.55 ± 1.17 | 3.563 ± 0.058 | 4.4+/-1.4 | 3.64+/-1.99 |

| Sample | Reduced Chi-square ($\chi_v^2$) | R-factor（%） |
|---|---|---|
| **$a_{g}$ZW-UiO-67·TFSA** | 567.41 | 0.0432 |

| amp/ $S_0^2$ | $N_{(Zr\text{-}O\ path)}$ | $R_{(Zr\text{-}O\ path)}$ (Å) | $\sigma^2_{(Zr\text{-}O\ path)}$ ($10^{-3}Å^2$) | $\Delta E_0$ (eV) |
|---|---|---|---|---|
| 1.03 | 6.92 ± 1.48 | 2.224 ± 0.088 | 3.1+/-1.7 | 2.18+/- 1.33 |
| amp/ $S_0^2$ | $N_{(Zr\text{-}Zr\ path)}$ | $R_{(Zr\text{-}Zr\ path)}$ (Å) | $\sigma^2_{(Zr\text{-}Zr\ path)}$ ($10^{-3}Å^2$) | $\Delta E_0$ (eV) |
| 0.82 | 3.46 ± 1.09 | 3.641 ± 0.093 | 4.8+/-1.9 | 4.35+/-2.02 |

| Sample | Reduced Chi-square ($\chi_v^2$) | R-factor（%） |
|---|---|---|
| **$a_{gr}$ZW-UiO-67·TFSA** | 551.89 | 0.0338 |

| amp/ $S_0^2$ | $N_{(Zr\text{-}O\ path)}$ | $R_{(Zr\text{-}O\ path)}$ (Å) | $\sigma^2_{(Zr\text{-}O\ path)}$ ($10^{-3}Å^2$) | $\Delta E_0$ (eV) |
|---|---|---|---|---|
| 1.03 | 7.71 ± 1.01 | 2.189 ± 0.079 | 3.2+/-1.0 | 2.24+/- 1.13 |
| amp/ $S_0^2$ | $N_{(Zr\text{-}Zr\ path)}$ | $R_{(Zr\text{-}Zr\ path)}$ (Å) | $\sigma^2_{(Zr\text{-}Zr\ path)}$ ($10^{-3}Å^2$) | $\Delta E_0$ (eV) |
| 0.82 | 3.52 ± 0.93 | 3.584 ± 0.096 | 4.5+/-1.9 | 4.49+/-1.96 |

**Supplementary Table 4 |** Atom-to-atom distances obtained from crystal structure of **ZW-UiO-67** and its calculated PDF data via PDFGui software, and those from experiment PDF plots of **ZW-UiO-67**, **$a_g$ZW-UiO-67·MSA** and **$a_g$ZW-UiO-67·TFSA** (see also Fig. 4a, Supplementary Fig. 22 and that following the Table).

| | **Atom—Atom** | **ZW-UiO-67** Crystal/Calc./Exp.(Å) | **$a_g$ZW-UiO-67·MSA Exp.(Å)** | **$a_g$ZW-UiO-67·TFSA Exp.(Å)** |
|---|---|---|---|---|
| **a**[a] | Zr—O | 2.10/2.05/2.16 | 2.34 | 2.19 |
| **b** | Zr—Zr | 3.49/3.46/3.46 | 3.55 | 3.45 |
| **c** | Zr—Zr | 4.95/4.88/4.75 | 4.95 | 4.76 |
| **d** | Zr—C | 6.30/6.28/6.25 | 6.63 | 6.27 |
| **e** | Zr—C | 8.49/8.53/8.39 | 8.79 (8.05) | 8.66 |
| **f** | Zr—C | 10.20/10.40/10.40 | 10.01 (10.69) | 10.31 |
| **g** | Zr—C | 12.70/12.70/12.50 | 12.05 (12.81) | 12.49 |
| **h** | Zr—Zr | 15.06/15.10/15.07 | 15.28 (14.51) | 14.81 |
| **i** | Zr—Zr | 17.07/17.05/16.67 | 17.71 | 17.13 (16.26) |
| **j** | Zr—Zr | 18.39/18.60/18.34 | 18.08 (19.07) | 18.46 |
| **k** | Zr—Zr | 20.36/20.54/20.25 | 20.28 | 20.22 |
| **l** | Zr—Zr | 22.51/22.30/21.83 | 22.58 | 21.72 |

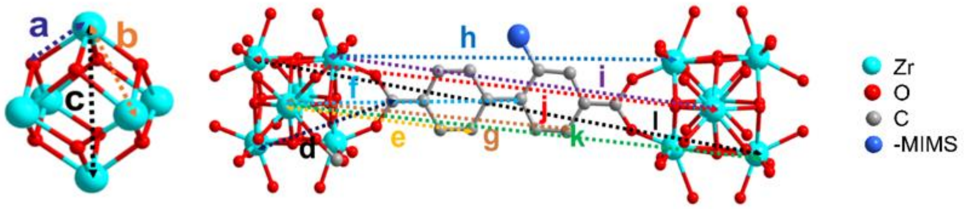


[a]Letters marked in Fig. 4a, Supplementary Fig. 22 and that following the Table.

**Supplementary Table 5 | Compared melting behaviors of HA/ILs@ZW-UiO-67** to that of **HA/ILs@UiO-67** (see also Supplementary Figs. 5-6, 29, Table 1,2, 4).

| | Meltability of ILs@ZW-UiO-67 and ILs@UiO-67 (heated to 320 °C) | | Meltability/$T_m$ (°C) of ZW-UiO-67·HA and HA@UiO-67 | | | |
|---|---|---|---|---|---|---|
| MOF \ ILs/HA | EMIM·TFS* | EIMS·MSA* | MSA | TFSA | TFA | ESA |
| ZW-UiO-67[a] | No | / | Yes /127.2 | Yes /149.6 | Yes /157 | Yes /139 |
| UiO-67[b] | No | No | No/- | No/- | No/- | No/- |
| ρ (g/ml)[c] | 1.387 | 1.42 | 1.48 | 1.892 | 1.696 | 1.388 |
| $pK_a$ | / | / | -1.9 | -12.2 | -14 | 1.8 |

* EMIM·TFS = 1-ethyl-3-methylimidazolium trifluoromethanesulfonate, EIMS = 1-(1-ethyl-3-imidazolium)propane-3-sulfonate. [a] For synthesis of **EMIM·TFS@ZW-UiO-6**7, de-solvated **ZW-UiO-**67 (80 mg) and 34 μL ILs (**EMIM·TFS**) (equal to the pore volume of MOF) were used. [b] For **ILs@UiO-67** and **HA@UiO-67**, activated UiO-67 (80 mg) and 81 μL (equal to the pore volume) HA or ILs were respectively used. All samples were obtained by the same incipient wetness technique (see also Method).

**Supplementary Table 6 |** Comparison of thermal behaviors of **ZW-MOF·HA** in this study.

| Samples | $\Delta H_{fusion}$(J/g) | $T_g$ (°C) | $T_m$ (°C) |
|---|---|---|---|
| **ZW-UiO-67·MSA** | 6.35 | 104 | 127 |
| **ZW-UiO-67·TFSA** | 4.28 | 122 | 152 |
| **ZW-UiO-67·TFA** | 12.4 | 110 | 157 |
| **ZW-UiO-67·ESA** | 11.45 | 82 | 139 |
| **ZW-DUT-5·MSA** | 7.78 | 65 | 152 |
| **ZW-UiO-68·MSA** | 8.15 | 98 | 156 |

*See also Fig.1, Supplementary Figs. 5, 6, 29, 32, 34 and Table 3.

## Supplementary References

1. Amarasekara, A. S. Acidic ionic liquids. Chem. Rev. 116, 6133–6183 (2016).

2. Cavka, J. H. et al. A new zirconium inorganic building brick forming metal organic frameworks with exceptional stability. *J. Am. Chem. Soc.* **130**, 13850–13851 (2008).

3. Li, S. et al. Mechanical properties and processing techniques of bulk metal-organic framework glasses. *J. Am. Chem. Soc.* **141**, 1027–1034 (2019).

4. Gotthardt, M. A. et al. Synthesis and post-synthetic modification of amine-, alkyne-, azide- and nitro-functionalized metal–organic frameworks based on DUT-5. Dalton Trans. 44, 16802–16809 (2015).
