# Supplementary material for: Melt-quenched glass formation of a family of metal-carboxylate frameworks
Source: Nat Commun. 2024 Mar 6;15:2040. doi: 10.1038/s41467-024-46311-x (PMC10917788; doi:10.1038/s41467-024-46311-x)
Supplement: Supplementary file 4 — Description of Additional Supplementary Files [file 41467_2024_46311_MOESM4_ESM.docx]

**Description of Additional Supplementary Information**

**Title:** Supplementary Movie 1

**Description:** Microscope video of *in-situ* heating of **ZW-UiO-67·MSA** from room temperature to 150 °C, demonstrating clear melt flow.

**Title:** Supplementary Movie 2

**Description:** Microscope video of *in-situ* heating of **ZW-UiO-67·TFSA** from room temperature to 150 °C, demonstrating clear melt flow.

**Title:** Supplementary Movie 3

**Description:** Microscope video of *in-situ* heating of **ZW-UiO-67·TFA** from room temperature to 165 °C, demonstrating clear melt flow.

**Title:** Supplementary Movie 4

**Description:** Microscope video of *in-situ* heating of **ZW-UiO-67·ESA** from room temperature to 145 °C, demonstrating clear melt flow.

**Title:** Supplementary Movie 5

**Description:** Microscope video of *in-situ* heating of **ZW-DUT-5·MSA** from room temperature to 155 °C, demonstrating clear melt flow.

**Title:** Supplementary Movie 6

**Description:** Microscope video of *in-situ* heating of **ZW-UiO-68·MSA** from room temperature to 140 °C, demonstrating clear melt flow.
